# Immunotoxins and Anticancer Drug Conjugate Assemblies: The Role of the Linkage between Components

**DOI:** 10.3390/toxins3070848

**Published:** 2011-07-14

**Authors:** Franco Dosio, Paola Brusa, Luigi Cattel

**Affiliations:** Department of Drug Science and Technology, University of Torino, Torino 10125, Italy; Email: paola.brusa@unito.it (P.B.); luigi.cattel@unito.it (L.C.)

**Keywords:** immunotoxin, antibody drug conjugate, linker, conjugation process, toxins, anticancer agents

## Abstract

Immunotoxins and antibody-drug conjugates are protein-based drugs combining a target-specific binding domain with a cytotoxic domain. Such compounds are potentially therapeutic against diseases including cancer, and several clinical trials have shown encouraging results. Although the targeted elimination of malignant cells is an elegant concept, there are numerous practical challenges that limit conjugates’ therapeutic use, including inefficient cellular uptake, low cytotoxicity, and off-target effects. During the preparation of immunoconjugates by chemical synthesis, the choice of the hinge component joining the two building blocks is of paramount importance: the conjugate must remain stable *in vivo* but must afford efficient release of the toxic moiety when the target is reached. Vast efforts have been made, and the present article reviews strategies employed in developing immunoconjugates, focusing on the evolution of chemical linkers.

## 1. Introduction

Cancer is becoming the most frequent cause of death in most developed countries, and in particular the incidence of this disease among women is increasing dramatically. In the US, the estimated number of new cancer cases was above 1.5 million in 2010, with a mortality rate accounting for 23% of total deaths in the USA [1].

Hundreds of approaches and strategies have been developed, and others (such as nanotechnology, pharmacogenomics, *etc.*, are still in their early stages, but the concept of selectively delivering potent chemotherapeutics to a tumor is an old and very challenging idea, with a solid rationale. However, a really delicate balance between activity against cancer cells and systemic toxicity must be achieved. On this basis macromolecules (such as polymers, polysaccharides, and proteins) have been proposed to carry the active moiety, because they offer some relevant advantages: it has been established that high-molecular-weight conjugates passively accumulate in tumor tissue, because of the enhanced permeability of those tissues and the retention (EPR) effect [2,3]. Unlike their low-molecular-weight counterparts, macro-molecular drugs often encounter significant permeability barriers in the majority of normal tissues. In contrast, the poorly-formed tumor vasculature around solid tumors is more permeable to macromolecules than is normal vasculature [4,5]. Furthermore, the small number of lymphatic vessels in tumor tissue allows these macromolecules to be retained in the interstitial space, increasing intratumoral drug concentrations 10–100 fold compared with the concentration produced by an equivalent dose of the drug given conventionally [6,7].

Conjugation of cytotoxic agents with macromolecules improves the pharmacokinetic profile, by decreasing the volume of distribution and prolonging the distribution and elimination phases [8]. Furthermore, the slow release of active drug from the carrier results in sustained high intratumoral drug levels and lower plasma concentrations of the active drug. 

Although some proteins can specifically deliver the linked drug to the affected area, only monoclonal antibodies (mAbs) possess perfect suitability in terms of selectivity and flexibility. Indeed, the recent successful development of monoclonal antibodies that target key components of biological pathways has expanded the range of treatment options for patients with several cancers. 

Antibody-based therapeutics are of growing significance in cancer therapy, as evidenced by the fact that 28 such drugs have now been approved for oncologic indications by the FDA, for marketing in the USA. Among them, eight had global market revenues of above US $1 billion, and the combined global revenues of all exceed US $50 billion [9]. The market for these therapeutics is the fastest growing sector in the pharmaceutical industry. Currently, there are hundreds of mAbs for oncologic use now in clinical development, and progress in the development of antibody-based therapeutics is dramatically increasing [10,11].

Despite the clinical success of therapeutic mAbs, naked antibodies, targeting cell surface tumor antigens expressed on carcinomas, are rarely curative of themselves, and most are administered in combination with chemotherapy. Antibodies alone have shown some success in extending the lives of cancer patients, but in many cases more potent agents are required to attempt complete eradication of the cancer mass. Many different agents have been conjugated, including traditional anticancer agents, cytotoxic natural products, phytotoxins, radioisotopes, bioactive proteins, enzymes that activate prodrugs of cytotoxic agents, and photosensitizers. In order to exploit the maximal effect, the inherent potency of the released drug must be sufficient to kill the tumor cell, even at low concentrations. To achieve significant cytotoxicity, very potent agents must be used. The suitable candidate as immunoconjugate payload is thus a compound that is too toxic for use as stand-alone chemotherapeutic.

To achieve an improvement selective potency, the conjugate should preferentially release the active agent in or around the tumor tissue. Thus the following components are essential: a targeting agent, a biodegradable linkage, and a bioactive potent anticancer agent. Several aspects of the rational design of active conjugates may be of interest. The mAb must be selected taking into account both its stability and its capability to be derivatized with drugs or toxins without losing activity and specificity, but must also consider the antigen target and its pathway. This is challenging, because antigen targets on cell surfaces are often present in limited numbers, and the internalization process for antigen-antibody complexes is frequently inefficient. An example of 'ideal' antigen is represented by HER2 (the target of T–DM1, see below). It is expressed in millions of copies on an HER2-positive cancer cell, whereas other tissues express low levels of HER2. It is internalized fairly quickly and, also very important, it does not get downregulated [12].

Another important aspect is the balance between mAb modification due to chemical processes and the molar loading of the drug, which can be tuned to achieve the desired potency. However, one of the major limiting factor in delivering is related to the repeated courses of therapy and the development of host immune responses to both the mAb and the toxin. For this reason, an extent of drug substitution, commonly 2–4 drugs per antibody, or a ratio of 1:1 for conjugate toxins, provides the best therapeutic window. The role of the linker is of fundamental importance, because, in addition to delivery efficiency, the stability of the drug to mAb linkage is a key factor in determining therapeutic potential [13]. The linker must be stable in the bloodstream, so as to limit the damage caused to healthy tissue by highly-active anticancer agents. Decomposition or decay would release the cytotoxin before it can be delivered to the target site. Furthermore, after internalization the drug must be completely and efficiently released in its active form.

Theoretically, with an antibody-drug conjugate, activation of the free drug can occur either intracellularly or extracellularly. Several strategies have been developed to selectively release the therapeutic agent from a conjugate. The principal mechanisms involve the use of spacers that are cleavable by proteolysis of enzymes overexpressed in the tumor tissue, or of acid-sensitive linkages cleavable under the acidic conditions present in tumors, endosomes, and lysosomes [14]. Furthermore, exploiting the tumor’s hypoxic environment [15], reduction reactions can be used to efficiently release active drug from the non-toxic prodrug [16]. Self-immolative spacers have also been developed, comprising drug, linker, and trigger. The tumor-specific cleavage reaction takes place between trigger and linker, to form a drug-linker derivative, which then degrades spontaneously by elimination or cyclization, to release the free drug [17], preferably inside the affected tissues. As a result, exposure of normal tissues is limited, which is potentially associated with a more favorable toxicity profile [18]. Finally, the method of conjugation, which determines the drug loading stoichiometry and homogeneity, has been shown to play a role not only in pharmacokinetics, but also in activity, potency, and tolerability. This review focuses on the role of conjugation processes and, in particular, on the chemical linkers, and on their evolution both for immunotoxins (IT) and antibody-drug conjugates (ADC), illustrating the main results (including clinical data, where available) that such research has produced.

## 2. Immunotoxins

Immunotoxins are protein-based therapeutics comprising at least two functional domains, one allowing them to bind specific target cells, and one that kills the cells following internalization. ITs were first postulated by Paul Ehrlich 100 years ago, and were envisaged as “magic bullets”. They became a reality following the development of monoclonal antibody technology, which provided the necessary targeting specificity. Immunotoxins have been armed with selected extremely-potent microbial and plant products. The majority of these toxins belong to the group of ribosome-inactivating proteins (RIPs).

Plants and fungi produce a number of molecules with defensive functions, to protect themselves against pathogens, such as microorganisms, and predators, such as insects. These defense proteins include ribosome-inactivating proteins, which are capable of inhibiting RNA translation. A broad spectrum of activities, encompassing antiproliferative, antitumor, immunomodulatory, antiviral, antifungal and anti-insect activities, have been attributed to these proteins. Recently, several reviews have appeared [19,20,21,22] regarding both holotoxins (RIPs type II) composed of a catalytic “A-chain” disulfide-bonded to binding “B-chains”, and “hemitoxins” (RIPs type I), such as gelonin, saporin, *etc.*, containing only a catalytic chain. The bacterial toxins Pseudomonas exotoxin (PE) and difteria toxin (DT) are single-chain proteins, containing both binding and catalytic domains. Both plant and bacterial toxins are able to bind to the cell surface, and can internalize into endosomes, translocate into the cytosol, and catalytically inhibit ribosomes, which kills the cell by apoptosis. Great efforts have been made in research into ITs as anticancer agents, based on the observation that a single molecule of toxin in the cytosol is sufficient to kill the cell [23].

Type II RIPs have been isolated from plants belonging to the Asteridae, Liliidae, Magnoliidae and Rosidae, the bulk belonging to the Asteridae [24]. They are divided into toxic type II RIPs and non-toxic type II RIPs. The toxic ones include ricin from *Ricinus communis*, abrin from *Abrus precatorius*, volkensin from *Adenia volkensii*, and modeccin from *Adenia digitata*. Nigrins from *Sambus nigra*, and ebulin from *S. ebulus* are non-toxic RIPs. Nigrin b has much higher cell-free translation inhibitory potency, but much lower *in vitro* cytotoxicity and *in vivo* toxicity, than does ricin, due to the replacement of Tyr 249 in ricin by Phe in ebulin 1. Agglutinin-I from *Abrus precatorius* seeds is a type II RIP, with greatly attenuated toxicity compared with abrin, another type II RIP isolated from the same seeds, due to replacement of Asn-200 in abrin with Pro-199 in agglutinin I [25].

Type I RIPs have been isolated, most often from seeds and sometimes from leaves and roots of plants belonging to the Asteridae, Caryophyllidae, Liliidae, Magnoliidae, and Rosidae, the greatest number being isolated from the Rosidae, which comprises Cucurbitacea, Euphorbiaceae and Fabaceae [24].

In the last twenty years, RIPs of new structure have been isolated from flowering plants and mushrooms [20]. Some of these RIPs possess a molecular mass in the vicinity of 20 kDa and an *N*-terminal amino acid sequence that is distinctly different from those of the 30-kDa type I RIPs, which often demonstrate remarkable homology to one another. Small RIPs with a molecular mass of 10 kDa or below have been purified from the seeds of several gourds, which are members of the Cucurbitaceae. These are characterized by an abundance of arginine and glutamate or glutamine residues. Mushrooms produce RIPs with various molecular masses [26,27]. The *N*-terminal sequences of some of the mushroom RIPs isolated to date are similar to one another, but others are widely dissimilar. These new RIPs generally exhibit biological activities similar to those of type I RIPs.

Type I RIPs with small molecular mass have been isolated from plants both in the Cucurbitaceae family and outside it [19]. Mushrooms and other fungi also produce type I RIPs. The *N*-terminal amino acid sequences of these low-molecular-mass single-chained plant type I RIPs and mushroom type I RIPs, are distinct from those of classical type I and type II RIPs from plants. The biological activities of these low-molecular-mass type I RIPs, which include, alongside translation-inhibitory, also *N*-glycosidase and antifungal activities, await full elucidation. The active site residues of RIPs are distinct from the antigenic site residues, and RIPs with fully-preserved biological activities, but with decreased immunogenicity, have been produced. Other modern anticancer approaches concerning toxins are based on transcriptional targeting and protease specific targeting (Protease activated toxins). In particular, gene encoding toxins are important candidates as suicide genes for cancer therapy. For a recent comprehensive review, see [28].

Several of the above-mentioned RIPs have been used for the synthesis of immunotoxins [29]; an overview of this kind of immunoconjugates and their main peculiarities follows with a particular focus on the main linker systems adopted for their conjugation.

**Figure 1:**
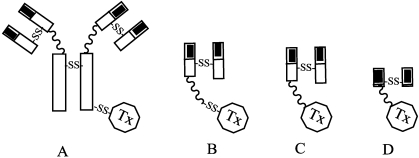
Structure of immunotoxins (IT) constructs obtained by chemical (A– intact IgG mAb, B– Fab’ fragment) and genetic engineering (C– Fab fragment, D– Fv fragment) procedures. TX as for toxin or fragment; white rectangles = constant regions, black rectangle = variable region of mAb chains; curvy linkage = peptide bond, –SS– = disulfide bond.

From the historical viewpoint, the evolution of approaches to constructing ITs may be subdivided into three stages. First-generation ITs were prepared by chemically linking to an antibody the whole molecule of a toxin, such as ricin, abrin, DT or PE. They were prepared by chemically conjugating antibodies to natural-intact toxin units or to toxins with attenuated cell binding capability (Figure 1).

Originally, antibodies were chemically conjugated through a disulfide bond, either to the whole toxin (holotoxins) or to their catalytic subunits (A chain), each of which had been removed from its binding domain by reduction. Many type I RIP have also been used to synthesize ITs; these toxins include gelonin, saporin, pokeweed antiviral protein (PAP), bryodin, bouganin, momordin, dianthin, momorcochin, trichokirin, lufﬁn, restrictocin, mitogillin, alpha-sarcin, Onconase, pancreatic ribonuclease, Bax, eosinophil-derived neurotoxin, and angiogenin. Among these, gelonin, saporin, momordin, PAP-s, and above all, ricin A chain were widely used to produce highly-active ITs, potentially avoiding aspecific toxicity [30].

**Figure 2:**
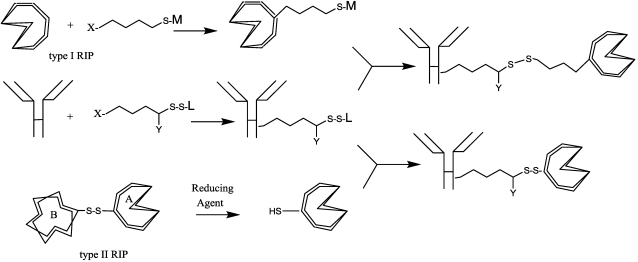
Scheme of reaction for synthesis of ITs. X = reacting group toward amino acid terminus; Y = H or alkyl, aryl group; M and L = leaving groups, stable in buffer but reactive in thiol-disulfide exchange; A and B = chains of RIP II toxins.

Chemical conjugates generally involve either reducible disulﬁde (S–S) or nonreducible thioether (S–C) bonds. A thioether bond is appropriate if the ligand is conjugated to a bacterial toxin in the part that does not translocate to the cytosol, such as the binding domain [31]. Otherwise, a disulﬁde bond is more commonly used (Figure 2).

One key aspect in creating an IT is that the linkage should be cleaved intracellularly, so as to regenerate the original active agent or a derivative of it, affording the cytotoxic action in full but, at the same time, conjugates must be stable *in vivo*: cleavage of the disulfide bond regenerates free antibody and toxin [32,33]. Premature cleavage reduces the amount of conjugate that can bind to target cells, inducing liver damage by free toxin. In addition, released antibody remains in circulation longer than the conjugate, and can compete with intact conjugate for target cell binding. Thus, in multiple-dose therapeutic treatment, the potency of the IT may decrease because tumor antigens are masked by the previously-released antibody.

Residues present on proteins involved in coupling with a crosslinker include primary amines, sulphydryls, carbonyls, carbohydrates and carboxylic acids. Conjugate preparation requires a separate derivatization of toxin and carrier with the linkers. Then, after purification, the newly-inserted group can react with those linked on the other IT component, to produce a stable and homogeneous conjugate population (Figure 2).

Different derivatizing approaches have been developed based on a covalent linkage. Several classes of crosslinking reagents have been synthesized, with the aim of improving the characteristics of the conjugates. Many factors must be evaluated in selecting an appropriate crosslinking reagent, including the existing groups present in the carrier or toxin, and the introduction of special reactive groups into agents. One important point to take into account is that the linkage method must be selected so as to avoid both the formation of homopolymers of antibody or agent, and aggregation of the conjugate.

**Figure 3:**
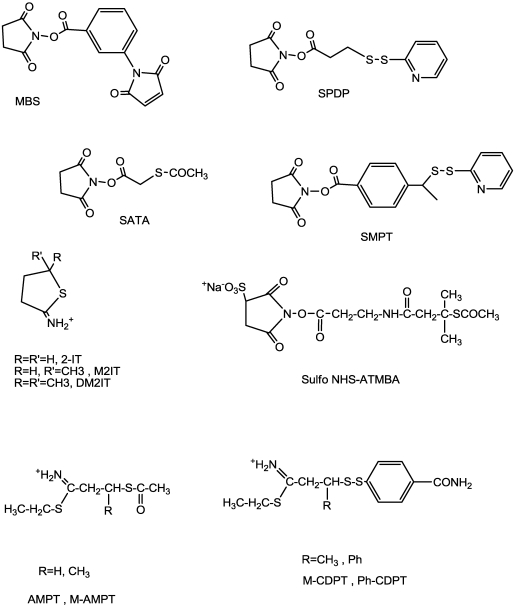
Scheme of heterobifunctional linkers used in conjugate preparations MBS, SPDP, SATA, 2-IT (2-iminothiolane) and linkers with improved hindrance around disulfide linkage. SMPT alpha-alkyl derivatives, SulfoNHS-ATMBA (Sulfosuccinimidyl *N*-[3-(Acetylthio)-3-methylbutyryl-beta-alanine]), and thioimidates AMPT, M-CDPT.

One of the first and most important heterobifunctional linkers is SPDP [*N*-succinimidyl 3-(2-pyridyldithio)propionate] (Figure 3), which reacts with the amino residues (terminal and lysines) of the protein, so that an activating disulfide can easily be inserted. This disulfide can then be reacted with the thiol group (added using the same reactive, after reduction, or using cysteine thiol of the previously reduced A chains) [34,35,36,37].

Other extensively-employed cross-linkers are SATA, [*S*-(*N*-succinimidyl) thioacetate], and SMPT, [(*N*-succinimidyloxy carbonyl)-1-methyl-1-(2-pyridyldithio) toluene], used to insert a disulfide linkage, while SMCC, [*N*-succinimidyl 4-(maleimidomethy1) cyclohexanecarboxylate] and MBS [3-maleimidobenzoic acid N-hydroxysuccinimide ester] afford the generation of a thioether linkage between moieties [38,39]. The main advantage of using SATA is the production of thiol by addition of a mild reagent (hydroxylamine) instead of a reducing agent (commonly dithiotreithol), which cannot react with native cysteine linkages [40].

In order to maintain the positive charge of lysyl groups also in the derivatized toxin, and thus to preserve their potency, reactives such as 2-iminothiolane (2-IT) or AMPT/CDPT have been used [32,41]. The usual procedure entails derivatization of the mAb with SPDP and 2-IT for the toxin [34].

Attempts to increase *in vivo* stability while maintaining high RIP activity have further focused on the synthesis of hindered cross-linking reagents, in which bulky side chains proximal to the disulfide bond afford protection from nucleophilic attack [38,41,42] (Figure 3). It has been shown that the presence of hindered disulfide linkage in ITs has little or no effect on their pharmacological potency, suggesting that disulfide cleavage is not the rate-limiting step in the intoxication of cells by conjugates. Furthermore, a significant enhancement of the pharmacokinetic profile (increased AUC) is directly related to the degree of steric hindrance.

As widely employed in prodrug approach, acid cleavable cross-linking reagents were also proposed for an efficient toxin release into endosomes and then in cytosol, avoiding translocation of the toxin into lysosomes and consequently complete denaturation. Blättler and colleagues described a heterobifunctional agent, which introduced a *cis*-aconityl bond, obtaining a conjugate with gelonin that was stable at neutral pH releasing active toxin in mildly acidic medium (pH 4.0–5.0). Based on this approach, conjugates between interleukin-2 (IL-2) and gelonin were synthesized with disulﬁde, acid-labile and noncleavable bonds [43]. Despite the desirable characteristics of acid-labile linkers, these reagents have not found further application in IT development.

The most commonly-employed type I RIP for ITs of clinical interest is ricin, because of its high cytotoxicity and low immunogenicity in man. Significant steps in the evolution of ITs have involved the use of deglycosylated RTA. However, several attempts have been made to maintain the high toxic potency of ricin, by reducing the non-specificity due to the presence of the B-chain. Thorpe *et al.* [44] developed a crosslinking method based on steric hindrance of the B chain, (using SPDP on mAb and *N*-hydroxysuccinimidyl ester of iodoacetic acid (SIA) on ricin) to shorten the thioether linkage to its limit (mAb-NHCO-CH_2_-CH_2_-SCH_2_-CONH-ricin), thus obtaining the so-called B-chain blocked ricin conjugates. The same approach has been successful using a smaller linkage (with reagents SATA and SIA) [45]. These conjugates have been tested *in vivo* in tumor mouse models, demonstrating improved specificity and potency. 

**Figure 4:**
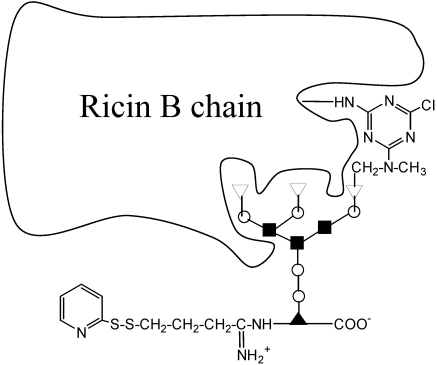
Scheme of preparation of blocked ricin. A triantennary *N*-linked oligosaccharide, present on glycopeptides from fetuin, is activated with cyanuric chloride (A chain is not shown).

Another extremely interesting approach is based on blockage of the B chain lectin binding ability; this was developed by Lambert (Immunogen). A glycopeptide containing a triantennary *N*-linked oligosaccharide is modified so as to covalently bind the receptor on B chain. The ligand is then activated at the 6-(*N*-methylamino)-6-deoxy-D-galactose residue, by reaction with cyanuric chloride. The resulting IT was found to be stable, and was defined as “blocked” (since ricin can no longer bind to a column of immobilized asialofetuin) and was shown to have aspecific toxicity that was 1000 times lower than the parent IT [46] (Figure 4). Another advantage of this approach is that these constructs are more homogeneous (only one linkage per ricin molecule) and more specific. Clinical trials of “blocked ricin” conjugates gave interesting early results, both in leukemias and in the therapy of small-cell lung cancer [47,48], nevertheless the studies on patients with chronic lymphocytic leukemia with minimal residual disease were suspended in 2003 [49] due to very limited activity of anti-B4 blocked ricin. The same results were obtained in untreated acute lymphoblastic leukemia [50].

The removal of nonspecific binding elements (B-chain in ricin/abrin) gave rise to the next generation of chemical conjugates, and broadened clinical applications. Immunotoxins were produced in a similar fashion, but thanks to the improved understanding of the structure and function of toxins, cell-binding domains were removed, and the resulting toxin fragment was targeted by coupling to an antibody. These ITs were also based on chemical conjugation between targeting moiety and toxin, and involved saporin, gelonin, PAP and deglycosylated RTA (dgA). Even without its binding domain, however, the ricin A chain is taken up non-specifically by macrophages and hepatic nonparenchymal Kupffer cells. This uptake is due to glycosylated side residues binding to mannose receptors on the liver. The most successful technique for reducing nonspecific uptake of the ricin A chain is through chemical deglycosylation [51]. Deglycosylated ricin A chain ITs show significantly prolonged lifetimes in mice, leading to an improved therapeutic index [39].

ITs containing dgA may be prepared by coupling the cysteinyl residue of deglycosylated ricin A-chain with the heterobifunctional crosslinker SMPT, that has previously been linked to mAb [52]. Using this approach, a stable but cleavable disulfide linkage was obtained, and aspecific recognition was reduced. Large-scale preparation (grams of IT) compliant with GLP has been described [53].

Diphtheria toxin (DT) is a 62 kDa protein secreted by *Corynebacterium diphtheria*. The single polypeptide chain contains an A chain, with ADP-ribosylation activity at the *N*-terminus, and a cell-binding domain at the *C*-terminus. *Pseudomonas aeruginosa* exotoxin A (PE) is a single peptide with three functional domains: domain Ia is the *N*-terminus and cell-binding domain; domain II has translocation activity; and domain III is the *C*-terminus and catalyses the adenosine diphosphate (ADP)-ribosylation. Immunotoxins composed of DT and PE were initially constructed by chemical synthesis [54,55] with the SPDP/2-IT method but, starting from 1990 these toxins were the base of interesting engineered contructs. Recombinant DT can be made by replacing the *C*-terminal cell-binding domain with a ligand that binds to a growth factor receptor, or to the Fv fragment of an antibody (Figure 1). Modification also reduces the non-specific binding of DT to human cells, thus increasing the toxin’s tumor-specificity 10,000-fold [56].

PE genetic excisions have concerned the domain Ia, generating a product termed PE 40, which retains its translocation function and protein synthesis inhibition properties, but does not kill human cells. A further genetically-engineered PE molecule (PE38KDEL) has been constructed by removing the disulfide bridge, and changing the carboxyl end of PE (KDEL) to increase its cytotoxic activity [57]. PE38KDEL has been fused with targeting moieties, for instance with the antibody Fv portion, or with other targeting agents, and the resulting molecule has been found to have a much higher affinity for binding to cancer cell lines than the native PE immunotoxin, and to be much more toxic to malignant cells [58].

Other modern constructs are represented by single-chain anti-Tac(Fv)-PE38 (LMB-2) that contains the variable heavy domain (VH) of an anti-Tac mAb fused via a peptide linker (G4S3) to the variable light domain (VL), which in turn is fused to PE38. On the other hand, the recombinant IT BL22 contains a disulﬁde bond connecting VL and VH by two inserted cysteine residues [58].

Several immunotoxins have been successfully tested in clinical trials for different purposes, and in particular in hematologic tumors. Anti-CD5 and anti-CD7, linked either to ricin or to chain A of ricin, have been used to eliminate contaminating tumor cells in autologous bone-marrow transplantants, and in acute graft *versus* host disease [59,60], non-Hodgkin’s lymphoma, and leukemias [59,61]. DT and PE constructs in the form of immunotoxins achieved better success, and have been evaluated in phase I trials in cancer patients [58,62,63]. Their extreme potency was demonstrated by Kreitman and Vitetta, in a study in which solid tumors in mice were eradicated like cells in tissue culture; they found that delivery of less than 1000 molecules/cell was sufficient to cause complete tumor regression [64].

Another factor influencing efficacy is immunogenicity: patients with antitoxin antibodies clear immunotoxins rapidly from the bloodstream. Since most people are immunized with DT, there is a significant pretreatment antibody titer in the blood of many patients, and an anamnestic response occurs in additional patients who have been treated with DT conjugates. Toxins that are foreign antigens to which a patient has not been previously exposed are of intermediate immunogenicity.

Another relevant aspect concerns the limits of the random-based derivatization approach. Although more specific, and thus better tolerated, most ITs are still chemically heterogeneous, and their large size hinders them from penetrating solid tumors. Moreover, some immunotoxins still bind weakly to normal cells, and produce an undesirable side effect known as vascular leak syndrome. To address these issues, a new generation of ITs was conceived and produced in the form of recombinant proteins. More successful IT design has employed genetic engineering, in which an amide bond, with or without a linker peptide, connects the mAb or its fragment to the toxin. Such fusions are more successful when both the receptor affinity and toxin domain functions can be preserved.

In the last eight years, using recombinant DNA techniques and the principles of protein engineering, ITs have been designed to contain only the elements required to recognize and kill the tumor cells. In particular, the remodeled agents of this generation are not only better at binding to receptors, but also at overcoming two major hurdles: toxicity and immunogenicity [65,66]. Most of the recombinant ITs currently in clinical trials use either DT or PE, because these bacterial toxins are more easily produced in *E. coli* than plant toxins, and have shown more activity and fewer side effects in humans. The mAb fragments are reduced to single-chain Fv, and recombinant ITs initially utilized single-chain Fv to target the toxin. Further, to increase stability a very stable Fv was designed, in which the peptide linker of the scFv was replaced by a disulﬁde bond, inserted in the framework region of the Fv [67]. Smaller and thus more diffusible contructs have been then made in large amounts and in homogeneous preparations within *E. coli*, giving the process more economically affordable.

Further improvement was presented by Keller *et al.* [68] with the principle of a molecular cleavable adapter that links the toxic moiety to the tumor-specific ligand. This molecular adapter contains three functional elements: a cytosolic cleavable unit, a cell penetrating peptide, and an endosomally cleavable unit. Further results suggest that cleavable adapters may be a useful tool in IT design, to reduce killing of nontargeted cells due to nonspecific binding [69].

Another way to target toxins to cells is to replace the cell-binding domain with a growth factor or a cytokine. The promising *in vitro* and *in vivo* activity of recombinant ITs or IL-2 and transferrin have led to advanced trials, and to the launch of Ontak (Denileukin Diftitox, Eisai) on the market (Table 1) for use against hematological malignancies and solid tumors among others [70,71,72]. 

**Table 1:** Immunotoxins in current status of clinical trials. Data from Thompson Pharma Partnering database and Clinicaltrials(March 2011), UTSMC—University of Texas Southwestern Medical Center.

**Immunotoxin Agent**	**Antigen Target**	**Toxic Component**	**Diseases**	**Clinical Phase**	**Company**
Ontak	IL-2R	DT	T-CLL, B-CLL, NHL	launched	Eisai
BL22	CD22	PE	Hairy Cell Leukemia, B-CLL, NHL	II	NCI
LMB-2	CD25	PE	NHL, leukemias	II	NCI
CAT-8015	CD22	PE	CLL, PLL, SLL	II	Medimmune
Combotox	CD19/CD22	dgA	Leukemias	I	Abiogen
HuM-195/rGel	CD33	r-Gelonin	Leukemias	I	Targa Ther.
MR1-1	EGFRvIII	PE	Solid ca.	I	Ivax Corp.
SS1P; CAT-5001	mesothelin	PE	Solid ca	II	NCI
Zemab	HER-2	PE	Breast ca	I	Novartis
RFT-5.dgA	IL-2R	dgA	lymphomas	II	UTSMC
Cintredekin besudotox	IL13R	PE	Brain ca	III	Insys

## 3. Antibody-Drug Conjugates

**Figure 5:**
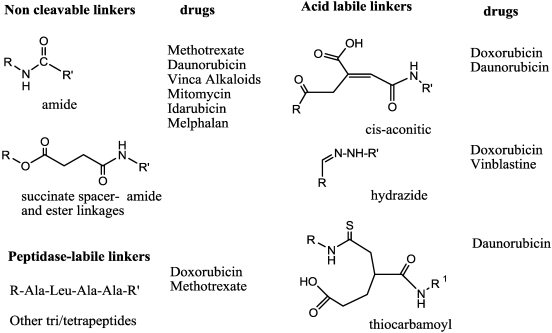
Examples of linker moieties.

First-generation antibody-drug conjugates involved the use of anticancer drugs in clinical use (see reviews by Chari *et al.* [73,74]). One of the key roles recognized early on was the nature of the linkage connecting mAb and toxic agent (which should be stable in blood circulation and should only release the free drug after internalization of the complex). In order to have release of free drug, acid labile linkers (labile around pH 5) and enzymatic labile linkers were chosen in particular (Figure 5); these released the drug by action of peptidase-esterase enzymes, thus in conditions present inside lysosomes but (e.g., esterases) also in the serum.

**Figure 6:**
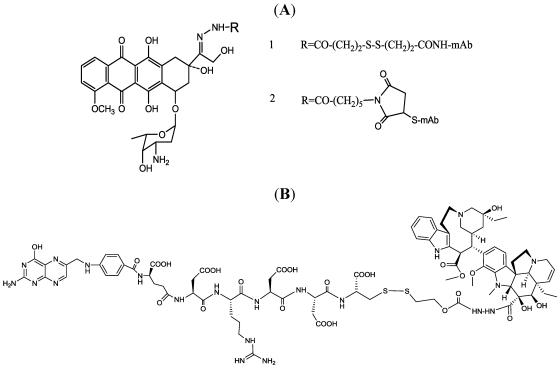
Examples of drug-linked conjugates with hydrazo bonds: (**A**) doxorubicin-mAb conjugate; (**B**) Vinca alkaloid bridge to a folate targeting moiety.

In the early development phase, methotrexate, daunorubicin, the Vinca alkaloids, mitomycin C, idarubicin and *N*-acetyl melphalan were linked through non-cleavable linkage (amide or using a succinimide spacer) to different murine mAbs [75,76,77,78,79,80,81]. The batches of drug produced were of a few mg (2–5 mg) and an average of 2–8 molecules per mAb were linked; in all cases, increasing the linkage lead to low conjugate yield. Although mAb recognition was maintained, the full potency of the drug was not, in these too-stable linkages. Further, two different approaches were then attempted, using peptide sequence lisosomally cleavable (-Leu-Ala-Leu-, Ala-Leu-Ala-Leu) [81,82] or an acid-labile link, such as cis-aconityl [83] or hydrazone bond. In particular, the cis-aconityl linkage was used also to join daunomycin to previously-oxidized and ethylendiamine-reacted carbohydrate groups [84]. The hydrazone linkage has been used to conjugate doxorubicin and vinblastine to mAbs. Doxorubicin was linked to the 13-keto group, and then to the mAb by a disulfide linkage [85] (Figure 6A1). In this case, release of the free drug required a disulfide reduction and/or acidic pH.

Trail *et al.* replaced the disulfide with a thioether linkage (Figure 6A2), maintaining the hydrazone between doxorubicin [86] and a BR96 mAb directed against a tumor-associated antigen closely related to Lewis Y (LeY). This ADC demonstrated high activity and impressive anti-tumor effects, including in well-established cancer models. This approach was also used to target other potent anthracyclines, such as 5-diacetoxypentyldoxorubicin and morpholinodoxorubicin [87], although the dose required to achieve a complete cure was very large (2 g/kg).

The use of these kinds of linkages (disulfide and hydrazone) deserves to be mentioned for its role in other relevant targeted approaches. Regarding doxorubicin, the maleimidocaproylhydrazone derivative (INNO-206) gave important results when administered as a prodrug, which could self-link to plasma albumin [88]. Regarding Vinca alkaloids, DAVLBH [89] (4-desacetylvinblastine-3-carbohydrazide) can be used as drug candidate to build a carbohydrate-linked mAb conjugate [90] based on a hydrazone linkage, which provides an improved therapeutic index *versus* the unconjugated drug. As for doxorubicin derivatives, a low-molecular-weight conjugate is also more promising: Endocyte have developed compound EC145, in which DAVLBH are linked through a disulfide bridge and a peptide spacer to the targeting agent folic acid [91] (Figure 6B). This compound at the time of publication is in phase III clinical trials.

The clinical results of the first-generation ADC were unsatisfactory, and, the reasons for these disappointing outcomes may be related to the early technological stage of the mAb in question (immune response, inefficient internalization), the lack of cytotoxic potency of the anticancer drug used, and to inefficient or premature release of the drug from the carrier. Premature drug release results in low specificity, low potency and an increase in the ADCs side effects.

Several reasons underlie the important results now being demonstrated by six second generation ADCs now in advanced clinical trials. Firstly, over the last 15 years, mAb technology has benefited from impressive improvements, and nine humanized or chimeric mAbs are now on the market for oncological use. The antibodies have been carefully chosen to improve selective binding to tumor tissue and to reduce cross-reactivity with healthy tissues. High avidity (Kd of 0.1 nm) in tumor binding has been reached. 

Furthermore, antibodies have been identified to antigens with high expression on the cell surface. For example, HER2 (one of the antigens employed formerly) presents favorable characteristics, namely high expression on HER2-positive cancer cells, rapid internalization, and no down-regulation.

The second important component of an ADC with improved action has been the selection of highly potent cytotoxic drugs. As yet, the laboratories of the main industries involved in ADC manufacturing (Immunogen, Seattle Genetics, Roche, Sanofi-Aventis, Pfizer) have selected only a few compounds, *i.e.*, auristatins, maytansines, and calicheamicin, which possess *in vitro* potency against tumor cell lines in the 0.01–0.1 nm range, 100–1000 fold more potent than first generation drugs. These compounds also show favorable physico-chemical characteristics, such as water solubility and stability. This is especially important to achieve adequate drug loading, without excessively modifying the mAb behavior.

The third component in modern ADC synthesis is the conjugation process itself. This has been improved in terms of linker/spacer moiety, conjugation specific site, and stoichiometry. The conjugation process should theoretically maintain the mAb and drug components in their native forms, and the product must remain intact during storage in aqueous solution or during the lyophilization/sterilization process, to allow convenient formulations. Furthermore, from the pharmaceutical development standpoint, the conjugation process must produce homogeneous batches and must be scalable, in order to satisfy the regulatory authorities. Several types of cleavable linkers have been evaluated, and the related approaches and evolution will be addressed below.

Regarding the mAb component, there are three common reactions for conjugation: alkylation of reduced interchain disulﬁdes, acylation of lysines, and alkylation of genetically-engineered cysteines. Because up to 100 lysines are available for acylation on one IgG1, conjugation to these sites results in heterogeneous mixtures. Eight interchain cysteines are available, and thus conjugates with a greater degree of uniformity than those based on lysine [92] can be obtained, while recombinant methods, in which cysteines are specifically introduced into the mAb backbone, provide even more uniform conjugates.

In some instances, it has been observed that the location of the conjugated drug is not as important as the stoichiometry of drug attachment [92,93]. ADCs with two to four drugs per antibody are generally superior to more heavily loaded conjugates, which clear very rapidly from the circulation [94]. In any case, using “random” chemical derivatization methods, it has proven difficult to prepare ADCs with only 2 drugs/mAb, because a large fraction of the mAb will not be conjugated to any drug.

The strategy concerning the toxic moiety and its ability to maintain high activity after derivatization requires an in-depth understanding of the organic-chemistry aspects of the process. Before illustrating the different synthetic approaches, it is necessary to have a clear view of the principal classes of toxic moiety.

### 3.1. Maytansinoids

Early clinical trials of maytansinoids initiated by the NCI in 1975, and their pre-clinical and phase I results prior to 1980, were summarized by Issel and Crooke [95]. These trials were carried out in patients with advanced disease, refractory to conventional therapy. Dose-limiting toxicity was established to be in the 1–2 mg/m^2^ range, and side effects included neurotoxicity, gastrointestinal toxicity, weakness, nausea, vomiting and diarrhea. The compound was evaluated over 35 tumors types but, based on poor clinical results and relevant side effects, the NCI closed the Investigational New Drug Application for maytansine used alone.

In the early 1990s, Takeda filed patents concerning the preparation of ADC using bispecific mAbs reactive to ansamitocins and to human transferrin receptors, that demonstrated effective activity in suppressing xenografted mouse tumor [96].

The ImmunoGen group took a chemical synthesis approach, which has led to the development of a number of conjugates [97,98].

**Figure 7:**
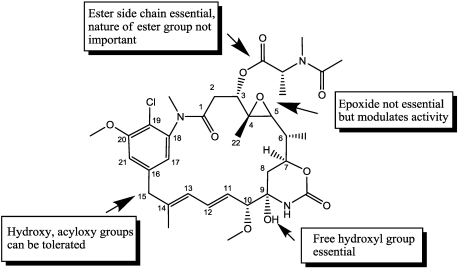
Some features of structure-activity relationship of Maytansine.

Although, as reported by Cassady [99] several chemical groups are not essential for inhibiting microtubule assembly, all conjugation procedures employed thus far have exploited the ester side chain (Figure 7).

The synthetic route to conveniently attain maytansine-analogues capable of being linked to mAbs started from ansamitocins obtained from fermentation of the microorganism *Actinosynnema pretiosum*. Then, with two steps (through maytansinol and the disulfide ester) reactive thiol-containing maytansinoids were obtained, as follows.

**Figure 8:**
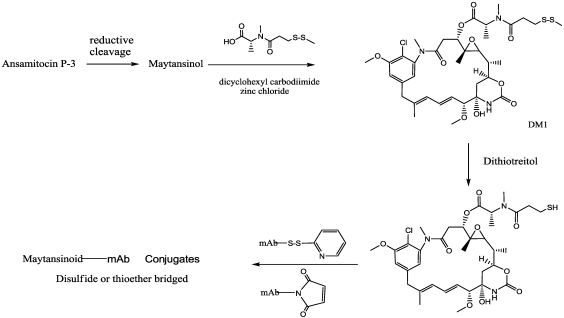
Synthesis of Maytansinoid-mAb conjugates.

Because the C3 ester group in maytansinoids is susceptible to elimination under mild basic conditions (pH > 9) giving the alpha-beta-unsaturated maytansinoid maysine, ester hydrolysis through a reductive cleavage process is employed. Initially, lithium aluminum hydride was used to give the C-3 alcohol maytansinol [100] but in a low yield. Successively , the ester group was efficiently cleaved using the mild reducing agent lithium trimethoxyaluminum hydride, under controlled temperature (−30 to −40 °C), to give the alcohol in good yields [101]. Maytansinol was then esterified with *N*-methyl-*N*-(methyldithiopropanoyl)-L-alanine in the presence of dicyclohexyl carbodiimide and zinc chloride, to give maytansinoid DM1 [100] (Figure 8).

After reductive cleavage of DM1 with dithiothreitol, the resulting thiol was widely conjugated to a variety of mAbs, including those directed against HER-2 and transferrin, [97] or cantuzumab (huC242-DM1) against mucin-type glycoprotein (CanAg), expressed to various extents by human colorectal cancers [98], or again, anti-CD44V6 (bivatuzumab mertansine [102]), huN901 licensed to Glaxo, trastuzumab-DM1 [103] MLN591-DM1 conjugate from Millenium, antiPSCA antibodies proposed as targeting agents for prostate cancer [104,105].

**Figure 9:**
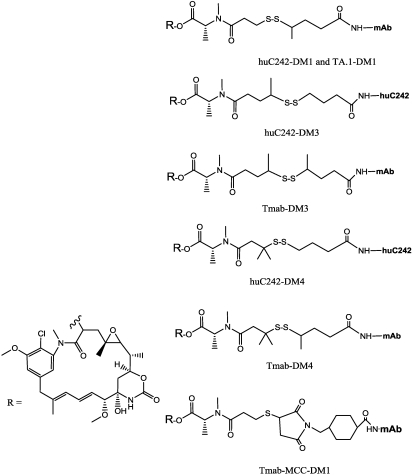
Maytansinoid conjugates with improved pharmacokinetics.

As described, this method have been used with success, but in order to increase the conjugate’s *in vivo* stability, allowing more specific intracellular release, the steric hindrance around the disulfide was examined. Widdison [101] described the synthesis of different ADCs bearing methyl groups placed around the disulfide. In the first huC242-DM1, the mAb was derivatized with SPP, or alternatively with SPDB. The derivatives DM3 and DM4 were obtained by *N*-Methyl-*N*-[(4-(R/S)-methyldithio)-1-oxopentyl]-*S*-alanine and *N*-Methyl-*N*-[(4-methyl-(4-methyldithio)-1-oxopentyl]-*S*-alanine (Figure 9). 

As mentioned above, the choice of disulfide bond release is an approach frequently employed in IT synthesis. This release is attributed to the high intracellular concentration of glutathione, a thiol-containing tripeptide present in micromolar concentrations in the blood, whereas its concentration in the cytoplasm is in the millimolar range (up to 1000 times higher). This is especially true for tumor cells, in which irregular blood flow leads to a hypoxic state, resulting in enhanced activity of reductive enzymes, and therefore even higher glutathione concentrations [106]. In the absence of sulfhydryl groups, disulfide bonds are thermodynamically stable and provide reasonably good stability in the bloodstream.

When tested in cytotoxicity trials, L-aminoacyl maytansinoid bearing a methyldithio-propanoyl substituent in the C3 ester side chain was found to be equally potent as maytansine. Furthermore, maytansinoids bearing sterically-hindered thiols were 3- to 12-fold more potent than un-hindered thiol derivatives. When tested in colon tumor xenograft models established in mice with COLO 205 cells, the ADC obtained with different disulfide hindrance showed different activities. 

*In vitro* cell tests demonstrated high specificity of the ADC, thus nontarget cells were not affected even at a 100-fold concentration. The huC242-DM3 exhibited relevant *in vivo* activity, more potent than DM1. This can be ascribed to release of the thiol-containing maytansinoid DM3, which is about 10-fold more potent than DM1, However, the greatest antitumor activity was that of huC242-DM4. This potency was attributed to at least two factors: (a) the greater stability of the disulfide bond between the antibody and the drug, which extends circulation time of the intact conjugate, and thus potentially produces greater accumulation at the tumor site; and (b) the greater stability of the released DM4 drug, which bears a thiol substituent at a tertiary carbon center.

**Figure 10:**
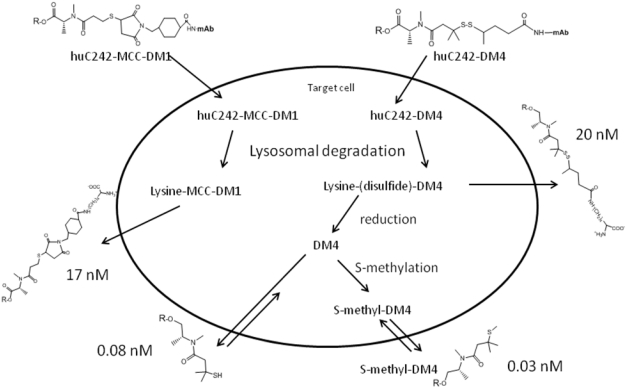
Model of metabolism and activation, of maytansine conjugates in a targeted cell. The number is referred to an IC50 value of maytansine derivatives on COLO205 cell line (given as an example).

The *in vivo* stability of the antibody–maytansinoid link, and the antitumor activities of two disulﬁde-linked huC242– maytansinoid conjugates (huC242–DM1 and huC242–DM4) and a thioether-linked conjugate (huC242–MCC–DM1), have also been compared in an *in vivo* model [107]. As predicted, the *in vivo* stability of the thioether-linked conjugate was the highest, with a half-life of 134 h, followed by huC242–DM4 (102 h). However, the conjugate huC242–DM4 showed the greatest efficacy in a human COLO 205 xenograft model in mice (see also Figure 10 and the S-methyl-D4 activity), suggesting a fine balance between the linker stability and antitumor activity.

Another preclinical study, in which trastuzumab was conjugated by mean of different linking systems to DM1, DM3 and DM4, reports results that are in contrast to these findings [108]. The stability and activity of the conjugates studied were tested by insertion of different methyl groups (one to three) around the disulfide bridge. All trastuzumab ADCs had average molar ratios of between 3 and 3.6 maytansinoid molecules per antibody. The pharmacokinetic profiles of MCC and DM4 derivative were similar, as was their antitumoral activity, although MCC was more active including in trastuzumab-resistant breast tumor models. Furthermore, more stable linkages were found to reduce *in vivo* toxicity (MCC corresponding to a DM1 dosage of 3264 μg/m^2^ had the same non-toxic effect as 653 μg/m^2^ of pure DM1, in rats). The thioether-linked trastuzumab-MCC-DM1 conjugate was found to have higher antitumor activity than any of the disulﬁde-linked conjugates, in mice bearing HER2-positive tumor xenografts, and in trastuzumab-refractory models (after a maximum of three doses of the maximal amount, *i.e.*, 420 μg/kg) (Figure 10).

**Figure 11:**
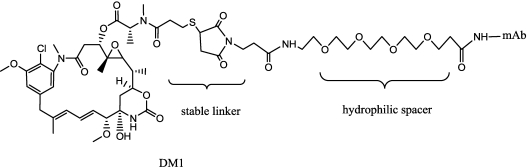
DM1-mAb conjugate with hydrophilic spacer.

Erikson [107,109] evaluated the metabolism of disulfide or thioether linked ADC in greater depth, clarifying particularly the role of ADC active metabolites. Three target-dependent tumor metabolites of the disulfide-linked huC242-DM4, namely lysine-Nε-(disulfide)-DM4, DM4, and *S*-methyl-DM4, were identified. The sole metabolite of the thioether-linked huC242-MCC-DM1 was lysineNε-MCC-DM1 (Figure 10). As expected, the (AUC for the metabolites of huC242-MCC-DM1 at the tumor over 7 days was about double that of the corresponding AUC for the metabolites of the disulfide-linked conjugates. The lipophilic metabolites of the disulfide-linked conjugates were found to be nearly 1000 times more cytotoxic than the lysineNε-MCC-DM1. This study predicted that the levels of the tumor metabolites accumulating *in vivo* would be high enough to allow for the metabolites to diffuse from the target cells within the solid tumors, providing support for the hypothesis that “bystander killing” contributes significantly to tumor eradication *in vivo*, upon treatment with cleavable disulﬁde-linked conjugates particularly for heterogeneously expressed targets like CanAg. The same research group, in a paper discussing the results obtained with trastuzumab DM1 conjugates [108], suggests that the distribution and delivery of maytansinoid metabolites may be sufficiently potent without the need for bystander killing, because the antibody target is sufficiently highly and homogeneously expressed throughout the tumor. To deeply evaluate the combination of linkage stability and mAb binding/internalization/trafficking, Polson *et al.* compared DM1 derivatives of a panel of seven mAbs the expression of which is largely restricted to the B-cell compartment and are expressed in the majority of non–Hodgkin’s lymphoma [110]. ADCs with cleavable linkers mediated *in vivo* efficacy via all these targets; ADCs with uncleavable (MCC) linkers were only effective when targeted to CD22 and CD79b expressed only in the B-cell compartment. The authors suggested that ADCs with cleavable linkers work on a broad range of targets, but for specific targets, ADCs with uncleavable linkers are superior with respect to safety. An improvement of the effects of affinity binding on microtubules and in the dynamic instability of maytansine metabolites (*S*-methyl-DM1 and *S*-methyl-DM4) was recently described by Lopus [111]. Although the lipophilic metabolites of maytansine show high potency, another more recent approach, involves the use of hydrophilic linker (or spacer) to bypass multi-drug resistance. Although MDR1 (permeability-glycoprotein transporter) has been shown to recognize and transport a great variety of compounds, most of the reported substrates are hydrophobic [112]. Kovtun *et al.* [113] described the synthesis and characterization of ADC composed of an anti–epidermal growth factor receptor (EGFR) antibody and maytansinoids, attached through a hydrophilic linker (Figure 11). The group hypothesized that the resulting hydrophilic metabolite (Lysine-derivative), might be a poor substrate of MDR1, thus avoiding MDR1-mediated resistance. The thioether linkage with DM1 may afford better stability and increased pharmacokinetics.

The results of trials of anti-EpCAM–PEG4-Mal–DM1 showed it to possess marked activity, not only on COLO 205 cells xenografted in mice, but also on a clone expressing a functional MDR1 pump, denoted COLO 205MDR, selected by culturing the cells in a medium containing paclitaxel. A single administered dose of 680 μg/kg as DM1 maintained the animals growth-free for 100 days after the implant. The group hypothesized that, although the metabolite, lysine-PEG Mal-DM1 was ∼8-fold less cytotoxic than the more lipophilic lysine-SMCC-DM1, mechanisms such as increased tubulin binding, inhibition of MDR1-mediated efflux, may play crucial roles in explaining the conjugate’s potency.

On the basis of promising preclinical results, several DM4 conjugates are now in advanced clinical trials. BT-062-SPDB-DM4 is in phase 2 trials for multiple myeloma [114]; also currently in trials are intetumumab-SPDB-DM4 (IMGN-388) from Centocor-Immunogen in a phase I trial for advanced solid tumors [115], SAR-3419 (HuB4-DM4), an anti-CD19 humanized monoclonal antibody conjugated to DM4, for the potential treatment of non-Hodgkin’s lymphoma, lorvotuzumab-MCC-DM1, (IMGN901) (anti CD56 mAb) (phase 2 for ovarian cancer small-cell lung cancer). and compound SAR-566658, comprising the monoclonal antibody DS6 which targets the Muc1epitope CA6 linked to DM4 from Sanofi-Aventis is in advanced preclinical trials [116]. Genentech has the conjugates trastuzumab-MCC-DM1 [103] (phase 2), RG-7593 (anti-CD22-MCC-MMAE) (phase 1 for non-Hodgkin’s lymphoma), and lorvotuzumab-DM1, (anti CD56 mAb) (phase 2) in advanced trials.

### 3.2. Dolastatins, Auristatin

Dolastatins are natural cytotoxic pseudopeptides extracted from the marine shell-less mollusk *Dolabela auricularia* (for an extensive review, see Pettit [117]). The dolastatin family has demonstrated antineoplastic, bactericidal, and fungicidal properties [118,119,120]. Within the family, dolastatin-10 and dolastatin-15 are potent disruptors of tubulin polymerisation [121], inhibit the binding of Vinca alkaloids to tubulin in a noncompetitive manner, and also stabilize the binding of colchicines to tubulin. Dolastatin 10 has demonstrated potent activity in preclinical studies, both *in vitro* and *in vivo*, against a range of lymphomas, leukemia and solid tumors [122]. Turner *et al.* [120] studied the effects of dolastatin-10 on the DU-145 human prostate cancer cell lines; they observed complete growth inhibition at concentrations of 1 nM. *In vivo* efficacy was demonstrated at a dose of 5mg of dolostatin-10, administered i.p. every 4 days in athymic mice. Further phase I clinical trials demonstrated dose limiting toxicities in the form of myelosuppression and phlebitis, with moderate peripheral neuropathy. Phase II trials have been carried out in non-small cell lung, prostate, melanoma, colorectal, ovarian, breast and pancreatobiliary tumors, but all have failed to demonstrate significant clinical activity in these tumors as a single agent [123]. With the aim to develop potent mAb- directed conjugates dolastatin 10 analogues, such as auristatin E (AE) and monomethylauristatin E (MMAE) have been selected [124,125,126], These compounds have been evaluated on a diverse panel of human tumor cell lines, including hematological malignancies, melanoma, and carcinomas of the lung, stomach, prostate, ovaries, pancreas, breast, colon and kidneys. The results indicated that none of the cell lines is resistant to AE, and that the drug (average half-maximal inhibitory concentration 3.2 ± 0.51 nm, 1 h exposure) is 200 times as potent as vinblastine [127].

**Figure 12:**
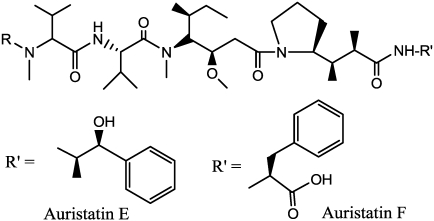
Structure of auristatins (R=CH_3_) and monomethylauristatins (R=H).

Different linking strategies have been evaluated, essentially employing two derivatization points of AE (starting from the left, *N*-or *C*-terminal position) (Figure 12); the more interesting strategy involves the use of acid-labile or proteolytically cleavable linkers. This approach is based on the characteristics of the intracellular route, through receptor-mediated endocytosis, and then through lysosomes that are both acidic and rich in highly active proteases.

**Figure 13:**
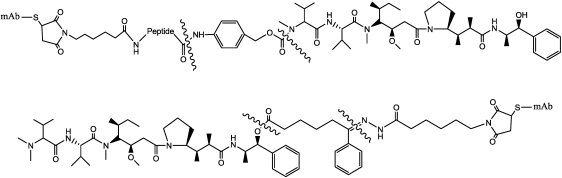
Structure of Auristatin E and MMAE conjugates. The wavy lines indicate the site of hydrolysis (enzymatic or pH-dependent).

Acid-labile linkers, containing hydrazone functionalities as the cleavable moiety, were formed at the *C*-terminus of AE by condensing maleimidocaproyl hydrazide with a panel of AE ketoesters. AEVB (Figure 13) was selected for additional studies because it is relatively stable at pH 7.2 (*t*^½^ > 60 h) but is labile at pH 5.0 (*t*^½^ 3 h). Protease-cleavable dipeptide linkers were attached to the *N*-terminal position of MMAE through a self-immolative *p*-aminobenzylcarbamate spacer (PABC) [127], and with the peptide linkers Phe-Lys- and Val-Cit-, that were quite stable under physiological conditions but underwent rapid hydrolysis in the presence of lysosomal extracts and purified human cathepsin B [128].

More recently, Doronina [129] explored the effects of linker technology on Auristatin F (AF)-based ADC potency, activity, and tolerability, by generating a diverse set of dipeptide linkers between the *C*-terminal residue. The group demonstrated that it is possible to alter the therapeutic window of auristatin-based ADCs, by changing the peptide linker between drug and mAb carrier protein. The *C*-terminal derivatives AF-Asn-(D)Lys and AF-Met-(D)Lys ADCs were not only more potent than Val-Cit-PABC-MMAF, but were also tolerated at higher doses. In this research, the linkage with mAb was achieved by its reduction with dithiothreitol, to expose the hinge cysteine thiol groups, followed by alkylation with the maleimido-containing MMAE and AEVB drug derivatives, forming conjugates with about eight drug molecules per mAb. This reductive conjugation method, although not often employed, is said by the study’s authors to preserve mAb affinity, lead to a high degree of conjugate uniformity, and provide yields in the range of 80% based on the mAb component.

Several ADCs containing auristatin are now in clinical trial; these are, from Seattle Genetics, Brentuximab vedotin (SGN-35) where an anti-CD30 monoclonal antibody (cAC10) is linked to Val-Cit-MMAE, SGN-75 composed by the anti-CD70 mAb 1F6 linked to Val-Cit-MMAF (phase I); from Celldex Ther. the conjugate glembatumumab, directed to melanoma antigen glycoprotein NMB, with Val-Cit-MMAE (CDX-011) (phase II) [130]; from Cytogen a mAb directed to prostate specific membrane antigen (PSMA) conjugated with Val-Cit-MMAE (PSMA-ADC; PSMA-ADC-1301) (phase I) [131] (for a recent review, see [132]). Very recently SGN-35 has obtained the Biologic License Application from FDA.

**Figure 14:**
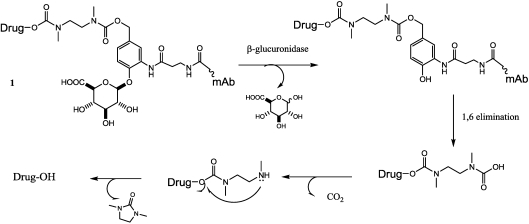
ADC composed of a glucuronidase activating linker (1) and the release mechanism.

In order to obtain linkers that can be efficiently and selectively cleaved intracellularly, maintaining a good *in vivo* stability, another approach has been attempted. Jeffrey *et al.* [133] describe the results of an approach to preparing ADCs comprising a β-glucuronide linker (Figure 14, compound 1) that they employed with several drug classes, such as auristatin [133], CBI minor groove binders [134], camptothecin and doxorubicin analogues, and very recently psymberin, a potent anticancer molecule with subnanomolar cytotoxic activity [135]. The mechanism of drug release requires cleavage by β-glucuronidase, an enzyme present in lysosomes and tumor interstitium [136]. The linker is highly stable in circulation and is hydrophilic; this allows it to be used with hydrophobic drugs that otherwise would lead to ADC aggregation.

### 3.3. Calicheamicins

The calicheamicins (also known as LL-E33288 antibiotics), produced by *Micromonospora echinospora ssp. calichensis*, were discovered by the American Cyanamid Co in 1986. These compounds are active in biochemical induction assays at concentrations below 1 pg/mL, extremely active against Gram-positive bacteria and also highly active against Gram-negative bacteria. Most interestingly, they show extraordinary potency against murine tumors: they are approximately 4000 times more active than adriamycin, with optimal dose at 0.5–1.5 μg/kg [137,138].

**Figure 15:**
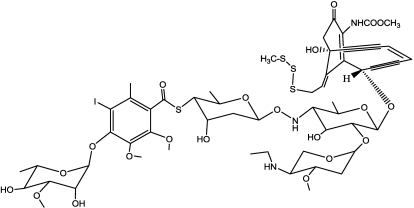
Structure of Calicheamicin gamma 1.

Calicheamicin gamma 1 (Figure 15) contains two distinct structural regions, each playing a specific role in the compound’s biological activity. The larger of the two regions consists of an extended sugar residue, comprising four monosaccharide units and one hexasubstituted benzene ring, which are joined together through a highly unusual series of glycosidic thioester and hydroxylamine linkages. The second structural region, the aglycon (termed calicheamicinone), contains a compact, highly-functionalized bicyclic core housing a strained enediyne unit within a bridging 10-member ring. The aryltetrasaccharide serves to deliver the drug to its target, tightly binding it to the minor groove of double-helix DNA.

**Figure 16:**
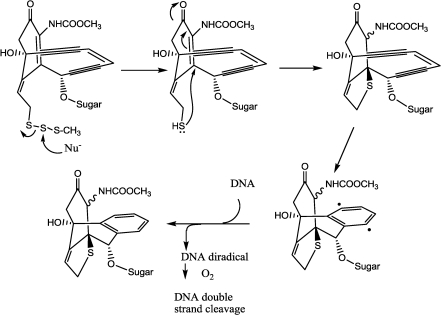
Mechanism of DNA cleavage by calicheamicin.

The aglycon is a rigid, highly-functionalized bicyclic core in which the enediyne moiety is locked within a rigid 10-member bridging ring awaiting activation. Also forming part of the aglycon is an allylic trisulfide, which serves as a trigger: when a nucleophile (e.g., glutathione) attacks the central sulfur atom of the trisulfide group, it causes a significant change in the structural geometry, which imposes considerable strain on the 10-member enediyne ring. This strain is completely relieved by the enediyne’s undergoing the cycloaromatization reaction, generating the highly-reactive 1,4-benzenoid diradical, that eventually leads to DNA cleavage [139] (Figure 16).

**Figure 17:**
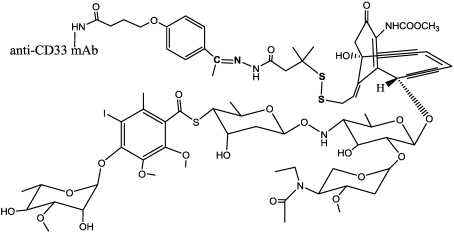
Structure of *N*-acetyl, gamma calicheamicin conjugate: Mylotarg.

A series of conjugates of calicheamicins with the CTM01 anti-MUC1 antibody has been reported [140] but the first and thus far the sole mAb linked to a cytotoxic payload that has been given regulatory approval is Wyeth’s gemtuzumab ozogamicin (Mylotarg; Figure 17) [141,142]. The drug was approved by the US Food and Drug Administration in year 2000 for use in patients over sixty suffering from relapsed acute myelocytic leukemia, the commonest form of leukemia in adults. Gemtuzumab ozogamicin consists of *N*-acetyl-γ-calicheamicin covalently attached to the humanized anti-CD33 IgG4 κ antibody (hP67.6) via a bifunctional linker. The 4-(4-acetylphenoxy)butanoic acid moiety provides attachment to surface-exposed lysines of the antibody through an amide bond, and forms an acyl hydrazone linkage with *N*-acetyl-γ-calicheamicin dimethyl hydrazide. Typically, a drug loading of 2 to 3 molecules of calicheamicin per molecule of mAb can be achieved. Upon internalization of the ADC, the calicheamicin prodrug is released by hydrolysis of the hydrazone in the lysosomes of the CD33+ target cells, at least *in vitro*. Indeed the hydrolysis of hydrazone linkage at 37 °C over 24 h increased from 6% at pH 7.4 to 97% at pH 4.5 [143]. The enediyne drug is then activated by reductive cleavage of the disulﬁde bond; and in order to prevent premature release of calicheamicin by circulating reduced thiols, such as glutathione, the disulﬁde linkage is stabilized by two methyl groups close to the disulfide.

In most preclinical models, the hydrazone linkage produces ADCs with higher potency than the corresponding amide-bearing conjugate, providing evidence that, with anti-CD33 mAb, the disulﬁde alone is insufficient for efficient release of the drug in the target cell. Interestingly, with the murine mAb CTM01 (recognizing the MUC1 antigen present on a broad spectrum of solid tumors of epithelial origin), amide-bearing ADC showed activities equal to or even greater than that of the corresponding hydrazone conjugate, in several *in vitro* and *in vivo* tumor models, revealing that this conclusion cannot be generalized at least without taking target internalization properties into account [144]. In clinical trials, however, the amide-bearing conjugate only showed limited evidence of activity.

Mylotarg enjoyed limited successes (and was withdrawn from the market in 2010) due to a narrow therapeutic window and lack of target-dependence. The linker technology based on a pH-dependent release mechanism is probably not sufficiently stable, and too much of the drug is released in the bloodstream; pharmacokinetic data have shown that the mean half-life of Mylotarg is 72 h [143]. Nonetheless, development of CMC544 (inotuzumab ozogamicin), which is a humanized anti-CD22 mAb identically attached to *N*-acetyl-γ-calicheamicin dimethylhydrazide via the acid-labile 4-(4′-acetylphenoxy)butanoic acid linker, is ongoing at Wyeth. Although this ADC is closely related to Mylotarg, the good stability shown in both human plasma and serum (rate of hydrolysis of 1.5–2%/day over 4 days) [144,145] make it more promising.

Regarding the role of production of more homogeneous batches of ADC, one of the key points is selectivity of the linkage insertion on the mAb molecule. As reported above, cytotoxic drugs are generally conjugated to antibodies either through lysine side-chain amines or through cysteine sulfhydryl groups activated by reducing interchain disulﬁde bonds. Both of these procedures yield of heterogeneous products, containing a mixtures of species with different molar ratios of drug linked to the antibody. Junutula pointed out the role of molecular biology in improving ADC using mAbs containing engineered cysteine residues for site-specific conjugation [146] (THIOMABs). In his first study, the chimeric (ch3A5) and fully-humanized (hu3A5) anti-MUC16 antibodies were engineered to have the HC-A114C mutation (Kabat numbering; equivalent to A118C in Eu numbering and A117C in sequential numbering) and conjugated with auristatin. In a following study, a THIOMAB version of trastuzumab (thio-TmAb) was designed, engineered at the cysteine residue at Ala114 (Kabat numbering), conjugated with DM1 [147]. The major advantage of this technique is the yield in homogeneous conjugate: in an improved process of ADC preparation (100 g of anti-MUC16 conjugate) more than 90% of the product was composed of 2 drug/mAb molecules.

**Figure 18:**
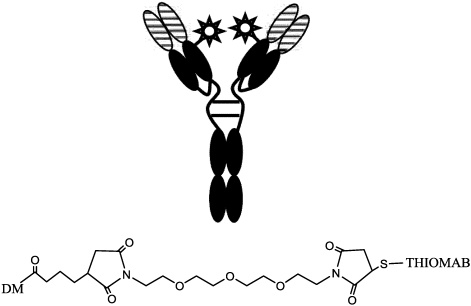
Scheme of specific insertion points on thio-trastuzumab and structure of the hydrophilic spacer.

In order to increase potency (as described above) and pharmacokinetic profile, thereby reducing systemic toxicity, a hydrophilic spacer and two non-reducible thioether linkages were applied. In this case, the homobifunctional reagent bis-maleimido-trioxyethylene glycol (BMPEO) was employed, reacted firstly with DM1, then with thio-trastuzumab (Figure 18).

This hydrophilic spacer is similar, as described above [113], but the engineered thioTmAb-mpeo-DM1 contained a precise stoichiometry and a specific linking site, on the single engineered cysteine residue on each heavy chain.

Engineered thioTmAb-MPEO-DM1 displayed equal *in vitro* activity and *in vivo* efficacy, at comparable antibody doses of conventional trastuzumab-MCC-DM1, but reduced aspecific toxicity, expressed as bone-marrow and liver toxicities. The elimination of high-drug-load ADC species produced an improvement in tolerability, *i.e.*, conjugate exposure associated with toxicity was greater for thioTmAb-MPEO-DM1 at 48 mg/kg than for trastuzumab-MCC-DM1at 30 mg/kg.

## 4. Conclusions

Starting from the chemical derivatization of toxins and mAbs, several conjugates have been developed, prepared with different approaches. Over the last 30 years, there has been continuous progress in IT design and development, leading to more sophisticated and efficacious products. Systemic off-target effects were prevalent when ITs were first introduced, but it is now possible to incorporate elements that reduce such effects, and to optimize the dose-response curves, so as to ensure the most potent possible effect on target cells, while limiting collateral damage. Molecular biology and protein engineering have been employed to develop potent and versatile toxin-based therapies with great application potential. Recent developments have reduced the size of ITs, allowing better distribution and penetration, improved binding affinity and avidity to cell-specific target antigens, and more efficient uptake and translocation to the cytosol. The potency, the reduction of immunogenicity, and the increase in specificity, have together improved the role of ITs as anticancer agents, although further human trials are needed: the true efficacy of these innovative products is still to be clarified, specifically their appropriateness for a narrow range of applications (e.g., interleukin-targeted cytotoxins in infusion techniques to treat brain cancer [148] or ITs for use in locoregional treatment [149]) or in a wider range of diseases.

Linker strategies took on great importance in the development of ADCs: synthesis is quite complex, several aspects must be critically balanced, and thus most ADCs providing promising preclinical data have failed to realize their potential in clinical trials. However, the latest generation of ADCs is now showing great promise in early clinical trials, particularly in hematological malignancies [145,150] With mature mAb technology, several potent compounds now under development that may be able to act on a wide variety of antigens, today’s solid knowledge of linker strategies, and the good variety of potent and well-known cytotoxic agents, the possibility to turn ADCs into effective and potent anticancer agents is truly beginning to take shape.

## References

[1] Cancer facts and figures. http://www.cancer.org/acs/groups/content/@epidemiologysurveilance/documents/document/acspc-026238.pdf.

[2] Greish K., Fang J., Inutsuka T., Nagamitsu A., Maeda H. (2003). Macromolecular therapeutics: Advantages and prospects with special emphasis on solid tumour targeting. Clin. Pharmacokinet..

[3] Maeda H. (2010). Tumor-selective delivery of macromolecular drugs via the EPR effect: Background and future prospects. Bioconjugate Chem..

[4] Jain R.K. (1987). Transport of molecules across tumor vasculature. Cancer Metastasis Rev..

[5] Roberts W.G., Palade G.E. (1997). Neovasculature induced by vascular endothelial growth factor is fenestrated. Cancer Res..

[6] Padera T.P., Kadambi A., di Tomaso E., Carreira C.M., Brown E.B., Boucher Y., Choi N.C., Mathisen D., Wain J., Mark E.J. (2002). Lymphatic metastasis in the absence of functional intratumor lymphatics. Science.

[7] Leu A.J., Berk D.A., Lymboussaki A., Alitalo K., Jain R.K. (2000). Absence of functional lymphatics within a murine sarcoma: A molecular and functional evaluation. Cancer Res..

[8] Cheng Y., Xu T. (2008). The effect of dendrimers on the pharmacodynamic and pharmacokinetic behaviors of non-covalently or covalently attached drugs. Eur. J. Med. Chem..

[9] Li J., Zhu Z. (2010). Research and development of next generation of antibody-based therapeutics. Acta Pharmacol. Sin..

[10] Reichert J.M. (2011). Antibody-based therapeutics to watch in 2011. MAbs.

[11] Reichert J.M. (2008). Monoclonal antibodies as innovative therapeutics. Curr. Pharm. Biotechnol..

[12] Hughes B. (2010). Antibody-drug conjugates for cancer: Poised to deliver?. Nat. Rev. Drug Discov..

[13] Ducry L., Stump B. (2009). Antibody-drug conjugates: Linking cytotoxic payloads to monoclonal antibodies. Bioconjugate Chem..

[14] Ulbrich K., Subr V. (2004). Polymeric anticancer drugs with pH-controlled activation. Adv. Drug Delivery Rev..

[15] De Groot F.M.H., Damen E.W.P., Scheeren H.W. (2001). Anticancer prodrugs for application in monotherapy: Targeting hypoxia, tumor-associated enzymes, and receptors. Curr. Med. Chem..

[16] Singh Y., Palombo M., Sinko P.J. (2008). Recent trends in targeted anticancer prodrug and conjugate design. Curr. Med. Chem..

[17] Damen E.W.P., Nevalainen T.J., van den Bergh T.J.M., de Groot F.M.H., Scheeren H.W. (2002). Synthesis of novel paclitaxel prodrugs designed for bioreductive activation in hypoxic tumour tissue. Bioorg. Med. Chem..

[18] Duncan R. (2006). Polymer conjugates as anticancer nanomedicines. Nat. Rev. Cancer.

[19] Stirpe F., Battelli M.G. (2006). Ribosome-inactivating proteins: Progress and problems. Cell. Mol. Life Sci..

[20] Girbés T., Ferreras J.M., Arias F.J., Stirpe F. (2004). Description, distribution, activity and phylogenetic relationship of ribosome-inactivating proteins in plants, fungi and bacteria. Mini-Rev. Med. Chem..

[21] De virgilio M., Lombardi A., Caliandro R., Fabbrini M.S. (2010). Ribosome-Inactivating proteins: From plant defense to tumor attack. Toxins.

[22] Ng T.B., Wong J.H., Wang H. (2010). Recent progress in research on ribosome inactivating proteins. Curr. Protein Pept. Sci..

[23] Yamaizumi M., Mekada E., Uchida T., Okada Y. (1978). One molecule of diphtheria toxin fragment A introduced into a cell can kill the cell. Cell.

[24] Battelli M.G. (2004). Cytotoxicity and toxicity to animals and humans of ribosome-inactivating proteins. Mini-Rev. Med. Chem..

[25] Bagaria A., Surendranath K., Ramagopal U.A., Ramakumar S., Karande A.A. (2006). Structure-function analysis and insights into the reduced toxicity of Abrus precatorius agglutinin I in relation to abrin. J. Biol. Chem..

[26] Conde F.P., Fernandez-Puentes C., Montero M.T.V., Vazquez D. (1978). Protein toxins that catalytically inactivate ribosomes from eukaryotic microorganisms Studies on the mode of action of alpha sarcin, mitogillin and restrictocin: Response to alpha sarcin antibodies. FEMS Microbiol. Lett..

[27] Ng T.B., Wang H.X. (2004). Flammin and velin: New ribosome inactivating polypeptides from the mushroom Flammulina velutipes. Peptides.

[28] Shapira A., Benhar I. (2010). Toxin-Based Therapeutic Approaches. Toxins.

[29] Bolognesi A., Polito L. (2004). Immunotoxins and other conjugates: Pre-clinical studies. Mini-Rev. Med. Chem..

[30] Pasqualucci L., Flenghi L., Terenzi A., Bolognesi A., Stirpe F., Bigerna B., Falini B. (1995). Immunotoxin therapy of hematological malignancies. Haematologica.

[31] Fitzgerald D., Idziorek T., Batra J.K., Willingham M., Pastan I. (1990). Antitumor activity of a thioether-linked immunotoxin: OVB3-PE. Bioconjugate Chem..

[32] Letvin N.L., Goldmacher V.S., Ritz J. (1986). *In vivo* administration of lymphocyte-specific monoclonal antibodies in nonhuman primates. *In vivo* stability of disulfide-linked immunotoxin conjugates. J. Clin. Invest..

[33] Blakey D.C., Watson G.J., Knowles P.P., Thorpe P.E. (1987). Effect of chemical deglycosylation of ricin A chain on the *in vivo* fate and cytotoxic activity of an immunotoxin composed of ricin A chain and anti-Thy 1.1 antibody. Cancer Res..

[34] Ebert R.F., Spryn L.A. (1990). Immunotoxin construction with a ribosome-inactivating protein from barley. Bioconjugate Chem..

[35] Cazzola M., Bergamaschi G., Dezza L., D’Uva R., Ponchio L., Rosti V., Ascari E. (1991). Cytotoxic activity of an anti-transferrin receptor immunotoxin on normal and leukemic human hematopoietic progenitors. Cancer Res..

[36] Scott C.F., Lambert J.M., Goldmacher V.S. (1987). The pharmacokinetics and toxicity of murine monoclonal antibodies and of gelonin conjugates of these antibodies. Int. J. Immunopharmacol..

[37] Bjorn M.J., Ring D., Frankel A. (1985). Evaluation of monoclonal antibodies for the development of breast cancer immunotoxins. Cancer Res..

[38] Thorpe P.E., Wallace P.M., Knowles P.P., Relf M.G., Brown A.N.F., Watson G.J., Knyba R.E., Wawrzynczak E.J., Blakey D.C. (1987). New coupling agents for the synthesis of immunotoxins containing a hindered disulfide bond with improved stability *in vivo*. Cancer Res..

[39] Ghetie V., Till M.A., Ghetie M.A., Tucker T., Porter J., Patzer E.J., Richardson J.A., Uhr J.W., Vitetta E.S. (1990). Preparation and characterization of conjugates of recombinant CD4 and deglycosylated ricin a chain using different cross-linkers. Bioconjugate Chem..

[40] Ghetie V., Vitetta E.S. (2001). Chemical construction of immunotoxins. Appl. Biochem. Biotechnol.: Part B Mol. Biotechnol..

[41] Arpicco S., Dosio F., Brusa P., Crosasso P., Cattel L. (1997). New coupling reagents for the preparation of disulfide cross-linked conjugates with increased stability. Bioconjugate Chem..

[42] Greenfield L., Bloch W., Moreland M. (1990). Thiol-containing cross-linking agent with enhanced steric hindrance. Bioconjugate Chem..

[43] McIntyre G.D., Scott C.F., Ritz J., Blattler W.A., Lambert J.M. (1994). Preparation and characterization of interleukin-2-gelonin conjugates made using different cross-linking reagents. Bioconjugate Chem..

[44] Thorpe P.E., Ross W.C.J., Brown A.N.F. (1984). Blockade of the galactose-binding sites of ricin by its linkage to antibody. Specific cytotoxic effects of the conjugates. Eur. J. Biochem..

[45] Cattel L., Delprino L., Brusa P., Dosio F., Comoglio P.M., Prat M. (1988). Comparison of blocked and non-blocked ricin-antibody immunotoxins against human gastric carcinoma and colorectal adenocarcinoma cell lines. Cancer Immunol. Immunother..

[46] Lambert J.M. (1991). The galactose-binding sites of the cytotoxic lectin ricin can be chemically blocked in high yield with reactive ligands prepared by chemical modification of glycopeptides containing triantennary *n*-linked oligosaccharides. Biochemistry.

[47] Collinson A.R., Lambert J.M., Liu Y., O’Dea C., Shah S.A., Rasmussen R.A., Goldmacher V.S. (1994). Anti-CD6-blocked ricin: An anti-pan T-cell immunotoxin. Int. J. Immunopharmacol..

[48] Grossbard M.L., Multani P.S., Freedman A.S., O’Day S., Gribben J.G., Rhuda C., Neuberg D., Nadler L.M. (1999). A Phase II study of adjuvant therapy with anti-B4-blocked ricin after autologous bone marrow transplantation for patients with relapsed B-cell non- Hodgkin’s lymphoma. Clin. Cancer Res..

[49] Tsimberidou A.M., Giles F.J., Kantarjian H.M., Keating M.J., O’Brien S.M. (2003). Anti-B4 blocked ricin post chemotherapy in patients with chronic lymphocytic leukemia—Long-term follow-up of a monoclonal antibody-based approach to residual disease. Leuk. Lymphoma.

[50] Szatrowski T.P., Dodge R.K., Reynolds C., Westbrook C.A., Frankel S.R., Sklar J., Stewart C.C., Hurd D.D., Kolitz J.E., Velez-Garcia E. (2003). Lineage specific treatment of adult patients with acute lymphoblastic leukemia in first remission with anti-B4-blocked ricin or high-dose cytarabine: Cancer and leukemia group B study 9311. Cancer.

[51] Blakey D.C., Thorpe P.E. (1986). Effect of chemical deglycosylation on the *in vivo* fate of ricin A-chain. Cancer Drug Delivery.

[52] Thorpe P.E., Wallace P.M., Knowles P.P., Relf M.G., Brown A.N.F., Watson G.J., Blakey D.C., Newell D.R. (1988). Improved antitumor effects of immunotoxins prepared with deglycosylated ricin A-chain and hindered disulfide linkages. Cancer Res..

[53] Ghetie V., Thorpe P., Ghetie M.A., Knowles P., Uhr J.W., Vitetta E.S. (1991). The GLP large scale preparation of immunotoxins containing deglycosylated ricin A chain and a hindered disulfide bond. J. Immunol. Methods.

[54] Bjorn M.J., Groetsema G., Scalapino L. (1986). Antibody-Pseudomonas exotoxin A conjugates cytotoxic to human breast cancer cells *in vitro*. Cancer Res..

[55] Thorpe P.E., Ross W.C.J. (1982). The preparation and cytotoxic properties of antibody-toxin conjugates. Immunol. Rev..

[56] Greenfield L., Johnson V.G., Youle R.J. (1987). Mutations in diphtheria toxin separate binding from entry and amplify immunotoxin selectivity. Science.

[57] Brinkmann U., Pai L.H., FitzGerald D.J., Willingham M., Pastan I. (1991). B3(Fv)-PE38KDEL, a single-chain immunotoxin that causes complete regression of a human carcinoma in mice. Proc. Natl. Acad. Sci..

[58] Kreitman R.J. (2001). Chimeric fusion proteins—Pseudomonas exotoxin-based. Curr. Opin. Invest. Drugs.

[59] Van Oosterhout Y.V.J.M., van Emst L., Schattenberg A.V.M.B., Tax W.J.M., Ruiter D.J., Spits H., Nagengast O.M., Masereeuw R., Evers S., de Witte T., Preijers F.W.M.B. (2000). A combination of anti-CD3 and anti-CD7 ricin A-immunotoxins for the *in vivo* treatment of acute graft *versus* host disease. Blood.

[60] Martin P.J., Pei J., Gooley T., Anasetti C., Appelbaum F.R., Deeg J., Hansen J.A., Nash R.A., Petersdorf E.W., Storb R. (2004). Evaluation of a CD25-specific immunotoxin for prevention of graft-versus-host disease after unrelated marrow transplantation. Biol. Blood Marrow Transplant..

[61] Schnell R., Borchmann P., Staak J.O., Schindler J., Ghetie V., Vitetta E.S., Engert A. (2003). Clinical evaluation of ricin A-chain immunotoxins in patients with Hodgkin’s lymphoma. Ann. Oncol..

[62] Kreitman R.J., Wilson W.H., White J.D., Stetler-Stevenson M., Jaffe E.S., Giardina S., Waldmann T.A., Pastan I. (2000). Phase I trial of recombinant immunotoxin anti-Tac(Fv)-PE38 (LMB-2) in patients with hematologic malignancies. J. Clin. Oncol..

[63] Pastan I., Hassan R., FitzGerald D.J., Kreitman R.J. (2007). Immunotoxin treatment of cancer. Annu. Rev. Med..

[64] Kreitman R.J., Pastan I. (1998). Accumulation of a recombinant immunotoxin in a tumor in vivo: Fewer than 1000 molecules per cell are sufficient for complete responses. Cancer Res..

[65] Li Y.M., Hall W.A. (2010). Targeted Toxins in Brain Tumor Therapy. Toxins.

[66] Onda M., Beers R., Xiang L., Nagata S., Wang Q.C., Pastan I. (2008). An immunotoxin with greatly reduced immunogenicity by identification and removal of B cell epitopes. Proc. Natl. Acad. Sci. USA.

[67] Benhar I., Pastan I. (1995). Identification of residues that stabilize the single-chain Fv of monoclonal antibodies B3. J. Biol. Chem..

[68] Keller J., Heisler I., Tauber R., Fuchs H. (2001). Development of a novel molecular adapter for the optimization of immunotoxins. J. Control. Release.

[69] Heisler I., Keller J., Tauber R., Sutherland M., Fuchs H. (2003). A cleavable adapter to reduce nonspecific cytotoxicity of recombinant immunotoxins. Int. J. Cancer.

[70] Dang N.H., Fayad L., McLaughlin P., Romaguara J.E., Hagemeister F., Goy A., Neelapu S., Samaniego F., Walker P.L., Wang M. (2007). Phase II trial of the combination of denileukin diftitox and rituximab for relapsed/refractory B-cell non-Hodgkin lymphoma. Br. J. Haematol..

[71] Gerena-Lewis M., Crawford J., Bonomi P., Maddox A.M., Hainsworth J., McCune D.E., Shukla R., Zeigler H., Hurtubise P., Chowdhury T.R. (2009). A phase II trial of denileukin diftitox in patients with previously treated advanced non-small cell lung cancer. Am. J. Clin. Oncol.: Cancer Clin. Trials.

[72] Kadin M.E., Vonderheid E.C. (2010). Targeted therapies: Denileukin diftitox-a step towards a magic bullet’ for CTCL. Nat. Rev. Clin. Oncol..

[73] Chari R.V.J. (2008). Targeted cancer therapy: Conferring specificity to cytotoxic drugs. Acc. Chem. Res..

[74] Chari R.V.J. (1998). Targeted delivery of chemotherapeutics: Tumor-activated prodrug therapy. Adv. Drug Delivery Rev..

[75] Endo N., Takeda Y., Kishida K., Kato Y., Saito M., Umemoto N., Hara T. (1987). Target-selective cytotoxicity of methotrexate conjugated with monoclonal anti-mm46 antibody. Cancer Immunol. Immunother..

[76] Pimm M.V., Paul M.A., Ogumuyiwa Y., Baldwin R.W. (1988). Biodistribution and tumor-localization of a daunomycin monoclonal antibody conjugate in nude-mice with human-tumor xenografts. Cancer Immunol. Immunother..

[77] Spearman M.E., Goodwin R.M., Apelgren L.D., Bumol T.F. (1987). Disposition of the monoclonal antibody-vinca alkaloid conjugate ks1/4-davlb (ly256787) and free 4-desacetylvinblastine in tumor-bearing nude-mice. J. Pharmacol. Exp. Ther..

[78] Kato Y., Tsukada Y., Hara T., Hirai H. (1983). Enhanced antitumor activity of mitomycin C conjugated with anti-alpha-fetoprotein antibody by a novel method of conjugation. J. Appl. Biochem..

[79] Rowland A.J., Pietersz G.A., McKenzie I.F.C. (1993). Preclinical investigation of the antitumor effects of anti-cd19-idarubicin immunoconjugates. Cancer Immunol. Immunother..

[80] Smyth M.J., Pietersz G.A., McKenzie I.F.C. (1987). Selective enhancement of antitumor-activity of n-acetyl melphalan upon conjugation to monoclonal-antibodies. Cancer Res..

[81] Trouet A., Masquelier M., Baurain R., Deprez-De Campeneere D. (1982). A covalent linkage between daunorubicin and proteins that is stable in serum and reversible by lysosomal hydrolases, as required for a lysosomotropic drug-carrier conjugate: *in vitro* and *in vivo* studies. Proc. Natl. Acad. Sci. USA.

[82] Umemoto N., Kato Y., Endo N., Takeda Y., Hara T. (1989). Preparation and *in vitro* cyto-toxicity of a methotrexate-anti-mm46 monoclonal-antibody conjugate via an oligopeptide spacer. Int. J. Cancer.

[83] Shen W.C., Ryser H.J. (1981). cis-Aconityl spacer between daunomycin and macromolecular carriers: A model of pH-sensitive linkage releasing drug from a lysosomotropic conjugate. Biochem. Biophys. Res. Commun..

[84] Dillman R.O., Johnson D.E., Shawler D.L., Koziol J.A. (1988). Superiority of an acid-labile daunorubicin monoclonal antibody immunoconjugate compared to free drug. Cancer Res..

[85] Greenfield R.S., Kaneko T., Daues A., Edson M.A., Fitzgerald K.A., Olech L.J., Grattan J.A., Spitalny G.L., Braslawsky G.R. (1990). Evaluation invitro of adriamycin immunoconjugates synthesized using an acid-sensitive hydrazone linker. Cancer Res..

[86] Trail P.A., Willner D., Lasch S.J., Henderson A.J., Hofstead S., Casazza A.M., Firestone R.A., Hellstrom I., Hellstrom K.E. (1993). Cure of xenografted human carcinomas by BR96-doxorubicin immunoconjugates. Science.

[87] King H.D., Staab A.J., Pham-Kaplita K., Yurgaitis D., Firestone R.A., Lasch S.J., Trail P.A. (2003). BR96 conjugates of highly potent anthracyclines. Bioorg. Med. Chem. Lett..

[88] Graeser R., Esser N., Unger H., Fichtner I., Zhu A., Unger C., Kratz F. (2010). INNO-206, the (6-maleimidocaproyl hydrazone derivative of doxorubicin), shows superior antitumor efficacy compared to doxorubicin in different tumor xenograft models and in an orthotopic pancreas carcinoma model. Invest. New Drugs.

[89] Schneck D., Butler F., Dugan W., Littrell D., Petersen B., Bowsher R., DeLong A., Dorrbecker S. (1990). Disposition of a murine monoclonal antibody vinca conjugate (KS1/4-DAVLB) in patients with adenocarcinomas. Clin. Pharmacol. Ther..

[90] Laguzza B.C., Nichols C.L., Briggs S.L., Cullinan G.J., Johnson D.A., Starling J.J., Baker A.L., Bumol T.F., Corvalan J.R.F. (1989). New antitumor monoclonal-antibody vinca conjugates LY203725 and related-compounds—design, preparation, and representative *in vivo* activity. J. Med. Chem..

[91] Dosio F., Milla P., Cattel L. (2010). EC-145, a folate-targeted Vinca alkaloid conjugate for the potential treatment of folate receptor-expressing cancers. Curr. Opin. Invest. Drugs.

[92] Sun M.M.C., Beam K.S., Cerveny C.G., Hamblett K.J., Blackmore R.S., Torgov M.Y., Handley F.G.M., Ihle N.C., Senter P.D., Alley S.C. (2005). Reduction-alkylation strategies for the modification of specific monoclonal antibody disulfides. Bioconjugate Chem..

[93] McDonagh C.F., Turcott E., Westendorf L., Webster J.B., Alley S.C., Kim K., Andreyka J., Stone I., Hamblett K.J., Francisco J.A., Carter P. (2006). Engineered antibody-drug conjugates with defined sites and stoichiometries of drug attachment. Protein Eng., Des. Sel..

[94] Hamblett K.J., Senter P.D., Chace D.F., Sun M.M.C., Lenox J., Cerveny C.G., Kissler K.M., Bernhardt S.X., Kopcha A.K., Zabinski R.F. (2004). Effects of drug loading on the antitumor activity of a monoclonal antibody drug conjugate. Clin. Cancer Res..

[95] Issell B.F., Crooke S.T. (1978). Maytansine. Cancer Treat. Rev..

[96] Okamoto K., Harada K., Ikeyama S., Iwasa S. (1992). Therapeutic effect of ansamitocin targeted to tumor by a bispecific monoclonal-antibody. Jpn. J. Cancer Res..

[97] Chari R.V.J., Martell B.A., Gross J.L., Cook S.B., Shah S.A., Blattler W.A., McKenzie S.J., Goldmacher V.S. (1992). Immunoconjugates containing novel maytansinoids: Promising anticancer drugs. Cancer Res..

[98] Liu C.N., Tadayoni B.M., Bourret L.A., Mattocks K.M., Derr S.M., Widdison W.C., Kedersha N.L., Ariniello P.D., Goldmacher V.S., Lambert J.M. (1996). Eradication of large colon tumor xenografts by targeted delivery of maytansinoids. Proc. Natl. Acad. Sci. USA.

[99] Cassady J.M., Chan K.K., Floss H.G., Leistner E. (2004). Recent developments in the maytansinoid antitumor agents. Chem. Pharm. Bull..

[100] Kupchan S.M., Sneden A.T., Branfman A.R., Howie G.A., Rebhun L.I., McIvor W.E., Wang R.W., Schnaitman T.C. (1978). Structural requirements for antileukemic activity among the naturally occurring and semisynthetic maytansinoids. J. Med. Chem..

[101] Widdison W.C., Wilhelm S.D., Cavanagh E.E., Whiteman K.R., Leece B.A., Kovtun Y., Goldmacher V.S., Xie H.S., Steeves R.M., Lutz R.J. (2006). Semisynthetic maytansine analogues for the targeted treatment of cancer. J. Med. Chem..

[102] Tijink B.M., Buter J., de Bree R., Giaccone G., Lang M.S., Staab A., Leemans C.R., van Dongen G. (2006). A phase I dose escalation study with anti-CD44v6 bivatuzumab mertansine in patients with incurable squamous cell carcinoma of the head and neck or esophagus. Clin. Cancer Res..

[103] Krop I.E., Beeram M., Modi S., Jones S.F., Holden S.N., Yu W., Girish S., Tibbitts J., Yi J.H., Sliwkowski M.X. (2010). Phase I Study of Trastuzumab-DM1, an HER2 antibody-drug conjugate, given every 3 weeks to patients with HER2-Positive metastatic breast cancer. J. Clin. Oncol..

[104] Ross S., Spencer S.D., Holcomb I., Tan C., Hongo J., Devaux B., Rangell L., Keller G.A., Schow P., Steeves R.M. (2002). Prostate stem cell antigen as therapy target: Tissue expression and *in vivo* efficacy of an immunoconjugate. Cancer Res..

[105] Helft P.R., Schilsky R.L., Hoke F.J., Williams D., Kindler H.L., Sprague E., DeWitte M., Martino H.K., Erickson J., Pandite L. (2004). A phase I study of cantuzumab mertansine administered as a single intravenous infusion once weekly in patients with advanced solid tumors. Clin. Cancer Res..

[106] Russo A., Degraff W., Friedman N., Mitchell J.B. (1986). Selective modulation of glutathione levels in human normal *versus* tumor-cells and subsequent differential response to chemotherapy drugs. Cancer Res..

[107] Erickson H.K., Park P.U., Widdison W.C., Kovtun Y.V., Garrett L.M., Hoffman K., Lutz R.J., Goldmacher V.S., Blättler W.A. (2006). Antibody-maytansinoid conjugates are activated in targeted cancer cells by lysosomal degradation and linker-dependent intracellular processing. Cancer Res..

[108] Phillips G.D.L., Li G.M., Dugger D.L., Crocker L.M., Parsons K.L., Mai E., Blattler W.A., Lambert J.M., Chari R.V.J., Lutz R.J. (2008). Trgeting HER2-Positive breast cancer with trastuzumab-DM1, an antibody-cytotoxic drug conjugate. Cancer Res..

[109] Erickson H.K., Widdison W.C., Mayo M.F., Whiteman K., Audette C., Wilhelm S.D., Singh R. (2010). Tumor delivery and *in vivo* processing of disulfide-linked and thioether-linked antibody-maytansinoid conjugates. Bioconjugate Chem..

[110] Polson A.G., Calemine-Fenaux J., Chan P., Chang W., Christensen E., Clark S., de Sauvage F.J., Eaton D., Elkins K., Michael Elliott J. (2009). Antibody-drug conjugates for the treatment of non-Hodgkin’s lymphoma: Target and linker-drug selection. Cancer Res..

[111] Lopus M., Oroudjev E., Wilson L., Wilhelm S., Widdison W., Chari R., Jordan M.A. (2010). Maytansine and Cellular Metabolites of Antibody-Maytansinoid Conjugates Strongly Suppress Microtubule Dynamics by Binding to Microtubules. Mol. Cancer Ther..

[112] Loo T.W., Clarke D.M. (2005). Recent progress in understanding the mechanism of P-glycoprotein-mediated drug efflux. J. Membr. Biol..

[113] Kovtun Y.V., Audette C.A., Mayo M.F., Jones G.E., Doherty H., Maloney E.K., Erickson H.K., Sun X., Wilhelm S., Ab O. (2010). Antibody-maytansinoid conjugates designed to bypass multidrug resistance. Cancer Res..

[114] Ikeda H., Hideshima T., Fulciniti M., Lutz R.J., Yasui H., Okawa Y., Kiziltepe T., Vallet S., Pozzi S., Santo L. (2009). The monoclonal antibody nBT062 conjugated to cytotoxic maytansinoids has selective cytotoxicity against CD138-positive multiple myeloma cells *in vitro* and *in vivo*. Clin. Cancer Res..

[115] Thompson D.S., Patnaik A., Bendell J.C., Papadopoulos K., Infante J.R., Mastico R.A., Johnson D., Qin A., O’Leary J.J., Tolcher A.W. (2010). A phase I dose-escalation study of IMGN388 in patients with solid tumors. J. Clin. Oncol..

[116] Al-Katib A.M., Aboukameel A., Mohammad R., Bissery M.-C., Zuany-Amorim C. (2009). Superior antitumor activity of SAR3419 to rituximab in xenograft models for Non-Hodgkin’s lymphoma. Clin. Cancer Res..

[117] Pettit G.R. (1997). The dolastatins. Fortschritte der Chemie Organischer Naturstoffe Progress in the Chemistry of Organic Natural Products Progrèss Dans La Chimie des Substances Organiques Naturelles.

[118] Mohammad R.M., Varterasian M.L., Almatchy V.P., Hannoudi G.N., Pettit G.R., Al-Katib A. (1998). Successful treatment of human chronic lymphocytic leukemia xenografts with combination biological agents auristatin PE and bryostatin 1. Clin. Cancer Res..

[119] Pettit G.R., Flahive E.J., Boyd M.R., Bai R., Hamel E., Pettit R.K., Schmidt J.M.  (1998). Antineoplastic agents 360. Synthesis and cancer cell growth inhibitory studies of dolastatin 15 structural modifications. Anti-Cancer Drug Des..

[120] Turner T., Jackson W.H., Pettit G.R., Wells A., Kraft A.S. (1998). Treatment of human prostate cancer cells with dolastatin 10, a peptide isolated from a marine shell-less mollusc. Prostate.

[121] Bai R., Pettit G.R., Hamel E. (1990). Binding of dolastatin-10 to tubulin at a distinct site for peptide antimitotic agents near the exchangeable nucleotide and vinca alkaloid sites. J. Biol. Chem..

[122] Kalemkerian G.P., Ou X., Adil M.R., Rosati R., Khoulani M.M., Madan S.K., Pettit G.R. (1999). Activity of dolastatin 10 against small-cell lung cancer *in vitro* and *in vivo*: Induction of apoptosis and bcl-2 modification. Cancer Chemother. Pharmacol..

[123] Krug L.M., Miller V.A., Kalemkerian G.P., Kraut M.J., Ng K.K., Heelan R.T., Pizzo B.A., Perez W., McClean N., Kris M.G. (2000). Phase II study of dolastatin-10 in patients with advanced non-small-cell lung cancer. Ann. Oncol..

[124] Miyazaki K., Kobayashi M., Natsume T., Gondo M., Mikami T., Sakakibara K., Tsukagoshi S. (1995). Synthesis and antitumor activity of novel dolastatin 10 analogs. Chem. Pharm. Bull..

[125] Pettit G.R., Srirangam J.K., Barkoczy J., Williams M.D., Durkin K.P.M., Boyd M.R., Bai R., Hamel E., Schmidt J.M., Chapuis J.C. (1995). Antineoplastic agents 337. Synthesis of dolastatin 10 structural modifications. Anti-Cancer Drug Des..

[126] Pettit G.R., Srirangam J.K., Barkoczy J., Williams M.D., Boyd M.R., Hamel E., Pettit R.K., Hogan F., Bai R.L., Chapuis J.C. (1998). Antineoplastic agents 365. Dolastatin 10 SAR probes. Anti-Cancer Drug Des..

[127] Doronina S.O., Toki B.E., Torgov M.Y., Mendelsohn B.A., Cerveny C.G., Chace D.F., DeBlanc R.L., Gearing R.P., Bovee T.D., Siegall C.B. (2003). Development of potent monoclonal antibody auristatin conjugates for cancer therapy. Nat. Biotechnol..

[128] Dubowchik G.M., Firestone R.A., Padilla L., Willner D., Hofstead S.J., Mosure K., Knipe J.O., Lasch S.J., Trail P.A. (2002). Cathepsin B-labile dipeptide linkers for lysosomal release of doxorubicin from internalizing immunoconjugates: Model studies of enzymatic drug release and antigen-specific *in vitro* anticancer activity. Bioconjugate Chem..

[129] Doronina S.O., Bovee T.D., Meyer D.W., Miyamoto J.B., Anderson M.E., Morris-Tilden C.A., Senter P.D. (2008). Novel peptide linkers for highly potent antibody-auristatin conjugate. Bioconjugate Chem..

[130] Younes A., Bartlett N.L., Leonard J.P., Kennedy D.A., Lynch C.M., Sievers E.L., Forero-Torres A. (2010). Brentuximab Vedotin (SGN-35) for Relapsed CD30-Positive Lymphomas. N. Engl. J. Med..

[131] Ma D., Hopf C.E., Malewicz A.D., Donovan G.P., Senter P.D., Goeckeler W.F., Maddon P.J., Olson W.C. (2006). Potent antitumor activity of an auristatin-conjugated, fully human monoclonal antibody to prostate-specific membrane antigen. Clin. Cancer Res..

[132] Alley S.C., Okeley N.M., Senter P.D. (2010). Antibody-drug conjugates: Targeted drug delivery for cancer. Curr. Opin. Chem. Biol..

[133] Jeffrey S.C., Andreyka J.B., Bernhardt S.X., Kissler K.M., Kline T., Lenox J.S., Moser R.F., Nguyen M.T., Okeley N.M., Stone I.J. (2006). Development and properties of beta-glucuronide linkers for monoclonal antibody-drug conjugates. Bioconjugate Chem..

[134] Jeffrey S.C., Nguyen M.T., Moser R.F., Meyer D.L., Miyamoto J.B., Senter P.D. (2007). Minor groove binder antibody conjugates employing a water soluble beta-glucuronide linker. Bioorg. Med. Chem. Lett..

[135] Jiang X., Garcia-Fortanet J., de Brabander J.K. (2005). Synthesis and complete stereochemical assignment of psymberin/irciniastatin A. J. Am. Chem. Soc..

[136] De Graaf M., Boven E., Scheeren H.W., Haisma H.J., Pinedo H.M. (2002). Beta-glucuronidase-mediated drug release. Curr. Pharm. Des..

[137] Lee M.D., Dunne T.S., Chang C.C., Ellestad G.A., Siegel M.M., Morton G.O., McGahren W.J., Borders D.B. (1987). Calichemicins, a novel family of antitumor antibiotics. 2. Chemistry and structure of calichemicin.gamma.1I. J. Am. Chem. Soc..

[138] Lee M.D., Dunne T.S., Siegel M.M., Chang C.C., Morton G.O., Borders D.B. (1987). Calichemicins, a novel family of antitumor antibiotics. 1. Chemistry and partial structure of calichemicin.gamma.1I. J. Am. Chem. Soc..

[139] Smith A.L., Nicolaou K.C. (1996). The enediyne antibiotics. J. Med. Chem..

[140] Hinman L.M., Hamann P.R., Wallace R., Menendez A.T., Durr F.E., Upeslacis J. (1993). Preparation and characterization of monoclonal-antibody conjugates of the calicheamicins: A novel and potent family of antitumor antibiotics. Cancer Res..

[141] Hamann P.R., Hinman L.M., Beyer C.F., Lindh D., Upeslacis J., Flowers D.A., Bernstein I. (2002). An anti-CD33 antibody-calicheamicin conjugate for treatment of acute myeloid leukemia. Choice of linker. Bioconjugate Chem..

[142] Hamann P.R., Hinman L.M., Hollander I., Beyer C.F., Lindh D., Holcomb R., Hallett W., Tsou H.R., Upeslacis J., Shochat D. (2002). Gemtuzumab ozogamicin, a potent and selective anti-CD33 antibody-calicheamicin conjugate for treatment of acute myeloid leukemia. Bioconjugate Chem..

[143] Van der Velden V.H.J., te Mervelde J.G., Hoogeveen P.G., Bernstein I.D., Houtsmuller A.B., Berger M.S., van Dongen J.J.M. (2001). Targeting of the CD33-calicheamicin immunoconjugate Mylotarg (CMA-676) in acute myeloid leukemia: *In vivo* and *in vitro* saturation and internalization by leukemic and normal myeloid cells. Blood.

[144] DiJoseph J.F., Dougher M.M., Kalyandrug L.B., Armellino D.C., Boghaert E.R., Hamann P.R., Moran J.K., Damle N.K. (2006). Antitumor efficacy of a combination of CMC-544 (inotuzumab ozogamicin), a CD22-targeted cytotoxic immunoconjugate of calicheamicin, and rituximab against non-Hodgkin’s B-cell lymphoma. Clin. Cancer Res..

[145] Wong B.Y., Dang N.H. (2010). Inotuzumab ozogamicin as novel therapy in lymphomas. Exp. Opin. Biol. Ther..

[146] Junutula J.R., Raab H., Clark S., Bhakta S., Leipold D.D., Weir S., Chen Y., Simpson M., Tsai S.P., Dennis M.S. (2008). Site-specific conjugation of a cytotoxic drug to an antibody improves the therapeutic index. Nat. Biotechnol..

[147] Junutula J.R., Flagella K.M., Graham R.A., Parsons K.L., Ha E., Raab H., Bhakta S., Nguyen T., Dugger D.L., Li G.M. (2010). Engineered Thio-Trastuzumab-DM1 Conjugate with an Improved Therapeutic Index to Target Human Epidermal Growth Factor Receptor 2-Positive Breast Cancer. Clin. Cancer Res..

[148] Puri S., Mahapatra A.K., Hussain E., Sarkar C., Sinha S., Joshi B.H. (2009). A review of studies on targeting interleukin 4 receptor for central nervous system malignancy. Curr. Mol. Med..

[149] Brown J., Rasamoelisolo M., Spearman M., Bosc D., Cizeau J., Entwistle J., MacDonald G.C. (2009). Preclinical assessment of an anti-EpCAM immunotoxin: Locoregional delivery provides a safer alternative to systemic administration. Cancer Biother. Radiopharm..

[150] Polson A.G., Ho W.Y., Ramakrishnan V. (2011). Investigational antibody-drug conjugates for hematological malignancies. Exp. Opin. Invest. Drugs.

